# The combined effect of institutional quality and capital flows on food and nutrition security and undernourishment in Sub-Saharan Africa

**DOI:** 10.1371/journal.pone.0275345

**Published:** 2022-10-10

**Authors:** Danny Cassimon, Olusegun Fadare, George Mavrotas

**Affiliations:** 1 Institute of Development Policy (IOB), University of Antwerp, Antwerp, Belgium; 2 School of Agriculture, Policy and Development, University of Reading, Reading, United Kingdom; University of Pecs Faculty of Humanities: Pecsi Tudomanyegyetem Bolcseszettudomanyi Kar, HUNGARY

## Abstract

Issues related to malnutrition, broadly defined, have received a growing attention in recent years, not only in connection with the Sustainable Development Goals but also recently with the unprecedented Covid-19 pandemic. At the same time, there exists a complex interaction between institutions, capital flows, and food and nutrition security that has received less attention in the relevant literature. In this paper we estimate a series of dynamic panel data models to examine the impact of institutional quality and capital flows on food security, nutrition security and undernourishment by using panel data for 25 SSA countries over the period 1996 to 2018. One of the key contributions of the paper is the use of both aggregate and disaggregated capital flows to examine the impact on both food and nutrition security, a dimension that has been surprisingly neglected in most of the relevant literature. We combine this with the interaction of various types of capital flows with an institutional quality index we constructed from various governance indicators to examine the impact of institutions on the overall nexus. Finally, we examine the impact not only on food and nutrition security but also on undernourishment. Our findings clearly demonstrate the importance of a heterogeneity approach and reflect on earlier work regarding the role of institutional quality in the overall nexus between external capital flows and various measures of food and nutrition security which leads, and as expected, to an interesting variation in the results obtained, depending on the type of capital flows and the interaction with the governance indicators.

## 1. Introduction

The Sustainable Development Goal 2 (SDG-2) to “end hunger, achieve food security and improved nutrition and promote sustainable agriculture” [1], has received a lot of attention in recent years as part of the 2030 Agenda. The post-2015 SDG-2 commitments towards achieving Zero Hunger by 2030 has increased aggregate foreign capital flows into the Sub-Saharan Africa (SSA) region, especially development aid, to support developmental projects and humanitarian relief [2]. At the same time, however, recent years have witnessed a significant rise in the number of food insecure and malnourished people in the region. In particular, the prevalence of undernourishment in the region is the highest in the world, rising sharply from 17.6% in 2014 to 19.1% in 2019, more than twice the average in the rest of the world [3]. Countries experiencing armed conflict in the region are also burdened by food insecurity and malnutrition [4, 5]. For example, Nigeria alone has witnessed about 180% increase in the number of undernourished people over the last decade as reported in the 2019 Africa regional overview of food security and nutrition. Furthermore, weak institutional capacity in many SSA countries undermines efforts to achieve optimal level of food and nutrition security as they significantly impact on economic development [6, 7].

Food insecurity and malnutrition more broadly in SSA result from myriad of problems associated with various factors [4], including low technological innovations and adoption in agricultural practices [8]. For example, the practice of animal husbandry has also played an important role in enhancing sustainable development and improving food security of agricultural communities, particularly in SSA countries. However, a recent important study on Ethiopia has shown that the majority of the rural communities do not have the right knowledge about the importance of crossbreed cattle in terms of their high yield and limited financial access is also a reason for low numbers of crossbreed cattle, thus affecting food security in these communities [9].

While factors such as climate change affect countries globally, countries with limited economic growth struggle to achieve significant development targets and food self-sufficiency without inputs from foreign aid and other capital flows. Studies on the impact of foreign capital flows in SSA, mainly in connection with foreign direct investment, official development assistance, remittances, and portfolio equity, have largely focused on developmental indicators such as human capital development, economic growth, and employment among others [6, 10, 11]. However, the impact of foreign capital flows on food and nutrition security has not received significant attention and this needs to improve. This is important in order to directly quantify the capital flows impact on food and nutrition security, given that factors such as economic growth and employment do not directly translate into good welfare for citizens [12]. The arguments remain that non-inclusive growth in SSA undermines poverty reduction [13] and increases food insecurity and nutrition inequities [14]. Similarly, workers may be faced with months of unpaid salary arrears or poor minimum wages due to bad governance [15], thus, leading to reducing household food access.

At the same time, the quality of governance in SSA determines the size and efficiency of investments [16] and the effectiveness of the region’s economy in achieving food and nutrition security. In this regard, good governance is construed as the process that leads to the provision of an enabling environment for investors to thrive and the judicious use of public resources for public interest, and this hinges on the six pillars of governance, namely control of corruption, government effectiveness, political stability, rule of law, voice and accountability, and regulatory quality. Various studies have also stressed the importance of balancing government policies with good governance for sustained growth and development in SSA [7, 17]. The consequences of bad governance in SSA have also been documented by various studies. For example, Zúñiga and Mullard [18] have shown that the effect of the Structural Adjustment Programs (SAPs) implemented by most African countries in the past to improve food security and nutrition in the region was stalled by issues surrounding poor governance, as corruption, political rent-seeking and inefficiencies tended to thrive in privatization processes. Callaghy [19], and Martinez and Kukutschka [20] also reveal how government officials co-opted foreign aid for personal purposes in developing countries. Specifically, all the above six pillars of governance were shown to hinder growth in the West Africa sub-region [17].

Against this background, in this paper we employ a panel data of 25 countries in SSA spanning the period 1996 to 2018 to examine the combined impact of institutions and capital flows on food and nutrition security and undernourishment in the region. The uniqueness of this study is that it links the different types of capital flows and institutional quality indicators with both food and nutrition security outcomes using three different measures for food and nutrition security and undernourishment for a substantial number of SSA countries and over a long time period. By doing so, we improve upon earlier work in this area [21]. Finally, we were able to identify a significant relationship between institutional quality, capital flows and food and nutrition security (and undernourishment) by employing the dynamic generalised method of moments (GMM) estimator thus, adding further confidence on previous evidence on this front for the region [22].

The rest of the paper is structured as follows: In section 2 we discuss earlier studies in this important research and policy area and in section 3 we discuss data issues and the empirical methodology employed in the paper. Section 4 focuses on the empirical findings emanating from the study whereas the final section concludes the paper.

## 2. Earlier studies

Agricultural production remains the bedrock of economic development, and food and nutrition security in many SSA countries as evidence in the high correlation between percent annual growth in agricultural value added and GDP in the region [23]. Between 2000 and 2018, the SSA food production value added (crop and livestock) increased by about 4.3% per year in real USD, while the rest of the world average over this period was 2.7% per year. However, using data from 49 countries in SSA between 2000 and 2018, Jayne and Sanchez (2021) [24] have shown that the agricultural growth rate experienced largely results from cropland expansion and not productivity. For example, while the average annual percent increase of area under cassava production was 3.09, yield was 0.13 between 2000 and 2018. This finding emphasizes that the real growth that translates to better well-being is a significant increase in productivity, which is also made possible by strong institutions. A study on Ethiopia has also stressed the importance of agricultural productivity in the whole process, including the role of the key determinants of agricultural productivity, such as domestic and international trade in agricultural products, in assuring food security [25].

Furthermore, between 2000 and 2018, countries that received foreign aid to support agricultural inputs such as seeds and fertilizer, were able to significantly increase yield in cereal (maize and rice) measured as metric tons per harvested hectare, more than countries with no such assistance [23]. This evidence is also supported by findings from the study by Orji et al. (2014) [26] who found that foreign capital inflows in six countries the West Africa Monetary Zone over the period from 1981 to 2010 had a positive impact on GDP in those countries, with ODA contributing significantly to growth in Sierra Leone and Ghana. There is also evidence that agricultural productivity growth has significant implications for institutional development [27], while the reverse is also true.

The link between institutional quality, capital flows, and food and nutrition security remains a complex one [28]. Previous studies examining this relationship have shown notable variations in findings due to different types of capital flows employed in the analysis and the measures of food and nutrition security used. Some authors have used a composite indicator that synthesizes three main indicators used by the FAO to measure food and nutrition security [29], while others have employed a composite indicator to measure governance quality [21]. Additionally, in a number of other studies, components of institutional quality are not considered as inputs into food and nutrition outcomes, thus paying little attention to the conditional hypothesis which suggests that foreign aid may affect food security only in countries with good governance, in the light of an influential literature linking foreign aid to governance quality in aid-recipient countries [30–32].

Furthermore, most studies focus only on the policies of government targeted directly or indirectly at improving food and nutrition security. Some notable studies similar to our study have, however, looked at the joint effects of governance quality and capital flows on food and nutrition security in a developing country context [21, 22, 33, 34]. Yet, some results lack internal validity due to the type of empirical strategy employed which failed to account for endogeneity in the overall relationship [35]. For example, Dhahri and Omri [28] examined the impact of foreign direct investment (FDI) and foreign aid in 16 SSA countries on food security and poverty reduction. By employing the system GMM estimator they show that while FDI has a positive impact on food security and poverty reduction only specific types of foreign aid have positive impacts on food security. However, they also found that the joint effect of FDI and foreign aid had a stronger impact on food security and poverty reduction. Focusing on Nigeria, Ogunniyi and Igberi [35] employed an ordinary least square estimator to show that FDI has no impact on real per capita income, which is a proxy for economic access to food.

In another study [33], it was found that aggregate foreign aid, and the components of aid to the agricultural sector have a positive effect on food security. However, the interaction between foreign aid and governance results to a negative effect on food security, suggesting that foreign aid may improve food security only in countries with good governance quality. Similarly, Petrikova [34] extended coverage beyond SSA and examined whether the impact of development aid on food security and nutrition is conditioned on the quality of governance and on the type of aid flows. By using GMM and two-stage least squares estimators and panel data for 85 developing countries, the author finds that foreign aid has a small positive impact on food security. However, while multilateral aid may have a positive effect on food security, bilateral aid, loans, and agricultural aid were more conditioned on the quality of governance.

Another fact that also deserves significant consideration is that food and nutrition insecurity can both be a cause and consequence of bad governance [36, 37]. Empirical evidence has shown that hungry people are less physically productive and have little regards for law and order, thus undermining government effectiveness [38]. In addition, malnutrition in young children reduces cognitive development and ultimately limits economic productivity later in life [39, 40] and weakens government institutions. Conversely, political instability and violence or terrorism are mostly associated with food insecurity [41]. For example, some SSA countries like Chad, the Democratic Republic of Congo, Sudan, Comoros, Central Africa Republic, Eritrea, Libya, and Somalia are at the bottom of government effectiveness index and with the highest number of undernourished populations in the region [42]. The above may present some methodological challenges in empirical work such as simultaneity and reverse causality, and only a few studies have accounted for it in the empirical analysis [21].

By employing the GMM estimator on a panel data for 15 countries in SSA, Ogunniyi et al. [21] examined the impact of remittances on food and nutrition security and showed that remittances independently have a positive impact on nutrition security. However, a stronger positive impact on food and nutrition security emerges when remittances are intersected with the governance index. The positive impact of the governance index is the outcome from the contribution of control of corruption, government effectiveness, political stability, and the rule of law, as they individually have a growth effect on food and nutrition security in SSA. Although aggregate capital flows to the SSA region have increased in recent years [43], the recent Covid-19 pandemic, that has also affected capital flows to developing countries including the SSA region [2, 44, 45], is another wake-up call for the region to strengthen its institutions and enhance domestic resource mobilization.

## 3. Data and empirical strategy

### 3.1. Data description and measurement

We use aggregate data from the World Development Indicators (WDI), Worldwide Governance Indicators (WGI), and FAOSTAT for 25 SSA countries over the period 1996 to 2018 (Table 1 provides relevant details). The countries selected and years covered were determined on the basis of data availability. Extracted from the data are three indicators for measuring food and nutrition security and variables that could explain these outcomes as guided by the literature. Specifically, we employ governance indicators and capital flows in SSA as the determinants of interest, while accounting for countries’ economic and demographic characteristics, and macroeconomic policies.

**Table 1:** Data sources and summary statistics of variables used in the empirical analysis.

Variable	Description	Source	Mean	Standard deviation
***Outcomes***				
AVFP	Average value of food production (constant 1$ per person) (3-year average)	FAOSTAT	168.695	64.511
ADESA	Average dietary energy supply adequacy (%) (3-year average)	FAOSTAT	108.013	12.239
Undernourishment-PoU	Prevalence of undernourishment (percent) (3-year average)	FAOSTAT	21.473	12.806
***Determinants***				
Control of corruption score	Control of corruption score	WGI	-0.534	0.714
Government effectiveness score	Government Effectiveness score	WGI	-0.612	0.671
Political stability score	Political Stability score	WGI	-0.571	1.061
Rule of law score	Rule of law score	WGI	-0.525	0.747
Voice and accountability score	Voice and Accountability score	WGI	-0.556	0.826
Regulatory quality score	Regulatory quality score	WGI	-0.512	0.623
Composite governance index (CGI)	Composite value of governance indicators	Authors	-1.334	1.784
Foreign Direct Investment (FDI)	Foreign direct investment net inflows (BoP, current US$)	WDI	7.40e+08	1.74e+09
Portfolio Equity (PE)	Portfolio equity net inflows (BoP, current US$)	WDI	1.46e+08	9.85e+08
Official Development Assistance (ODA)	Net official development assistance received (current US$)	WDI	7.91e+08	1.15e+09
Remittances	Personal remittances received (current US$)	WDI	1.22e+09	4.19e+09
Capital flows (CF)	Mean value of all capital flows	Authors	7.25e+08	1.65e+09
CF x CGI	Interaction of logged composite governance indicator and logged capital flows	Authors	2.986	8.652
Share of agriculture in GDP	Agriculture, forestry, and fishing, value added (% of GDP)	WDI	21.915	14.656
Population growth	Percentage of population growth (annual %)	WDI	2.312	0.976
Inflation	Consumer price index (annual %) as proxy for inflation	WDI	8.422	15.592
Secondary school enrollment	Secondary school enrollment (% of gross) as proxy for human capital	WDI	34.497	16.636

Note: Countries included in the panel: Angola, Botswana, Burkina Faso, Cape Verde, Central Africa Republic, Côte d’Ivoire, Ethiopia, Gabon, Ghana, Lesotho, Madagascar, Malawi, Mali, Mauritius, Mozambique, Namibia, Niger, Nigeria, Rwanda, Senegal, Sierra Leone, South Africa, Swaziland, Togo, Uganda.

#### 3.1.1. Food and nutrition security measurement

The indicators used to capture food and nutrition security as obtained from FAOSTAT have already been aggregated. They include the average value of food production-AVFP, average dietary energy supply adequacy-ADESA, and undernourishment in a population (PoU). The AVFP shows the net food production value of a country, while ADESA consists of the ratio between the average caloric supply and the population’s actual needs. The PoU is the share of the population that is undernourished (people suffering hunger), i.e., those whose caloric intake is insufficient. It is a lead indicator for measuring hunger for international hunger targets such as the SDG-2, and a good proxy for the availability and access dimensions of food security at the country level. This measure captures the dimension of food and nutrition security situation of a country or a region. The three indicators employed here have also been used to proxy national food and nutrition security in the literature [46, 47].

#### 3.1.2. Governance indicators and capital flows measurement

The WGI is a set of composite indicators of six dimensions of governance for over 200 countries and territories since 1996 which allows for meaningful cross-country and over-time comparisons [48]. The six dimensions include (1) voice and accountability, (2) political stability and absence of violence/terrorism, (3) government effectiveness, (4) regulatory quality, (5) rule of law, and (6) control of corruption. They capture key aspects of governance which include political, economic, and legal aspects. Given the possibility of correlation between the six governance indicators and multicollinearity in an empirical model, we compute a composite governance index (CGI) from the six governance indicators using a principal component analysis approach. Capital flows from four main external sources are also employed in this study. These include Foreign Direct Investment (FDI), Portfolio Equity (PE), Official Development Assistance (ODA), and Remittances. In addition, we compute the mean of aggregate capital inflows from these sources over the same period.

#### 3.1.3. Other explanatory variables

We use three variables to capture a country’s economic and demographic characteristics which include the share of agriculture in GDP, population growth, and human capital formation, while the consumer price index-CPI (inflation) is used to capture the effect of country’s macroeconomic policies. These variables have been computed in the WDI and FAOSTAT databases. The share of agriculture in GDP is measured from the contributions of agriculture including crops and livestock, forestry, and fishing. When the contributions are driven by innovation and technological progress, they have the capacity to boost household food security, increase aggregate food supply and drive economic growth in SSA region [49]. Furthermore, the annual population growth rate is employed to account for the demographic change in SSA which is on the increase and at a faster pace than the aggregate food supply [50], thus, exerting a demographic pressure on the economy as food needs increase and per capita food availability decreases. We proxy human capital formation by using enrolment in secondary schools [51, 52]. Inflation rate measures macroeconomic stability and high inflation is associated with bad macroeconomic policies [53, 54]. While domestic stabilization policies that create an economically stable environment tend to have welfare enhancing effects, macroeconomic instability is found to increase poverty with undesirable effects on food and nutrition security [55, 56]. Table 1 shows the description of the variables used in the study and the relevant data sources.

### 3.2. Empirical strategy

By using a dynamic estimation approach, we analyze the impact of governance (both as a composite indicator and as disaggregated indicators) and capital flows (both as aggregate inflows of FDI, PE, ODA, and Remittances as well as disaggregated flows) on food and nutrition security in SSA from 1996 to 2018. We specify the dynamic panel model in order to account for the potential endogeneity that may arise from estimating the dynamic attributes of capital flows and governance. The appropriateness of this model is premised on the assumption that economic process is dynamic in nature. This is understandably true given that policy reforms potentially do have long-term impacts, spanning from the immediate into the future. In view of this, some studies using panel data have employed a dynamic approach in estimating relationships of this nature [21, 28, 45]. In a similar fashion, we specify the dynamic Eq (1) below for modelling food and nutrition security level as a function of past food and nutrition security level (a control for past policy reforms), and current factors.

The long-run effect of our control variables is accounted for by using the lagged dependent variable. The framework in Eq (1) assumes a Cobb-Douglas constant returns to scale production function in which the dependent and the explanatory variables are transformed into logarithmic forms, an approach that has been widely established in the relevant literature [57].

$$Y_{it}={\delta Y}_{it-1}+{\beta G}_{it}+{\rho C}_{it}+{\varphi X}_{it}+\gamma_{i}+\vartheta_{t}+\mu_{it}$$ (1)

where *Y*_*it*_ represents the food and nutrition security of country *i* at time *t* captured as AVFP, ADESA, and PoU. *Y*_*it*−1_ represents AVFP, ADESA, and PoU lagged one year. The governance indicators are captured in vector *G*_*it*_, where the governance index (CGI) is measured as the first principal component of the six governance indicators. *C*_*it*_ is a vector of capital flows. Governance and capital flows are treated as endogenous due to the possibility of reverse causality that may arise between the governance quality, capital flows, and the food and nutrition security outcome variables, given that countries may experience improved quality of governance and reduce capital flows in response to past food and nutrition security shocks. Vector *X*_*it*_ represents some control variables that also determine food and nutrition security of a country. Represented in *γ*_*i*_ *is* unobserved country-specific effects such as geographical and institutional factors that do not change with time; *ϑ*_*t*_ denotes the time-specific effect which accounts for shocks that do not vary among countries such as global demand shocks while *μ*_*it*_ is the error term. *δ*, *β*, *ρ*, *φ* are the estimated parameters. The subscripts *i* and *t* represent the country and time periods, respectively.

Having a lagged dependent variable with time invariant unobserved heterogeneity (*γ*_*i*_) in Eq (1) poses two key problems. One issue arises because *γ*_*i*_ is time invariant and results in serial correlation and inconsistent estimation if not accounted for, particularly if correlated with the explanatory variables. Another issue arises because *Y*_*it*_ is a function of *γ*_*i*_, and since it is also true for *Y*_*it*−1_, it is correlated with *μ*_*it*_ and bias OLS estimation upward [58]. Using the fixed-effects model is a common approach to solving these issues as it relies on the within changes over time for each country. However, Nickell [59] argued that fixed-effects model is limited in solving this problem given that the lagged dependent variable (*Y*_*it*−1_) is correlated with *μ*_*it*_. Also, demeaning the data is a common approach in the literature; however, this also results in biased estimates as the lags of the explanatory variables would be correlated with the demeaning variable [60]. Other methods to remove time invariant term and the unobservable heterogeneity include first differencing the data [61–63] and using forward orthogonal deviations [64]. In this paper we employ the first difference of the data. Hence, we rewrite Eq (1) as:

$$Y_{it}-Y_{i,t-1}=\delta (Y_{i,t-1}-Y_{i,t-2})+\beta (B_{it}-B_{i,t-1})+(\vartheta_{t}-\vartheta_{t-1})+({\mu_{it}}_{it}-{\mu_{it}}_{i,t-1})$$ (2)

where *B*_*it*_ includes *G*_*it*_, *C*_*it*_ and *X*_*it*_. Given that the error term of Eq (2) $\left({\mu_{it}}_{it}-{\mu_{it}}_{i,t-1}\right)$ is now correlated with the lagged dependent variable (*Y*_*it*−1_−*Y*_*it*−2_), instruments are required to address this problem. The instruments employ the panel nature of the data which consist of previous observations of the lagged dependent variable. Using this procedure, we account for potential endogeneity of other explanatory variables *G*_*it*_, *C*_*it*_ and *X*_*it*_. Given the assumptions that our error term is not serially correlated, and our explanatory variables are weakly exogenous, we use the lagged levels of the explanatory variables as instruments in our specification [65]. According to Arellano and Bond [63], the differencing approach alongside using the level of past values as instruments is referred to as the Difference-Generalized Method of Moments (Difference-GMM) estimator. This approach also has its own shortcomings. First, by taking the first-differences, information related to the long-run relationship between the explanatory variables and the dependent variable can be lost. Second, the lagged levels are shown to be weak instruments for first differences if the series are very persistent [66]. This may affect the asymptotic and small-sample performance of the Difference-GMM estimator [67].

To increase the efficiency of the Difference-GMM estimator, Arellano and Bover [64] suggest adding the original equation in levels to the system and referred to this as System-GMM estimator. Hence, we use the two-step System-GMM estimator featuring Windmeijer’s [68] finite-sample correlation for standard errors. The two-step System-GMM estimator employees an optimal weighting matrix for the moment conditions. To satisfy the consistency of the of the GMM estimator, we use the Arellano-Bond AR(1) and AR(2) tests of the serial correlation properties, and the Hansen [69] J-test of over-identifying restrictions. By using this we validate the assumption that lagged values of the explanatory variables are valid instruments.

## 4. Results and discussion

### 4.1. Descriptive results

#### 4.1.1. Correlation matrix of variables

The correlation matrices examine the pattern of the relationships between the regression models’ explanatory variables. Results are presented in Tables A1-C5 in the S1 Appendix. Correlation coefficients among the explanatory variables across the different specifications are less than 0.50, which is weak enough to suggest a potential problem of multicollinearity in our empirical analysis.

#### 4.1.2. Results of the bivariate relationship between food and nutrition security, undernourishment, and governance and capital flows

**Fig 1:**
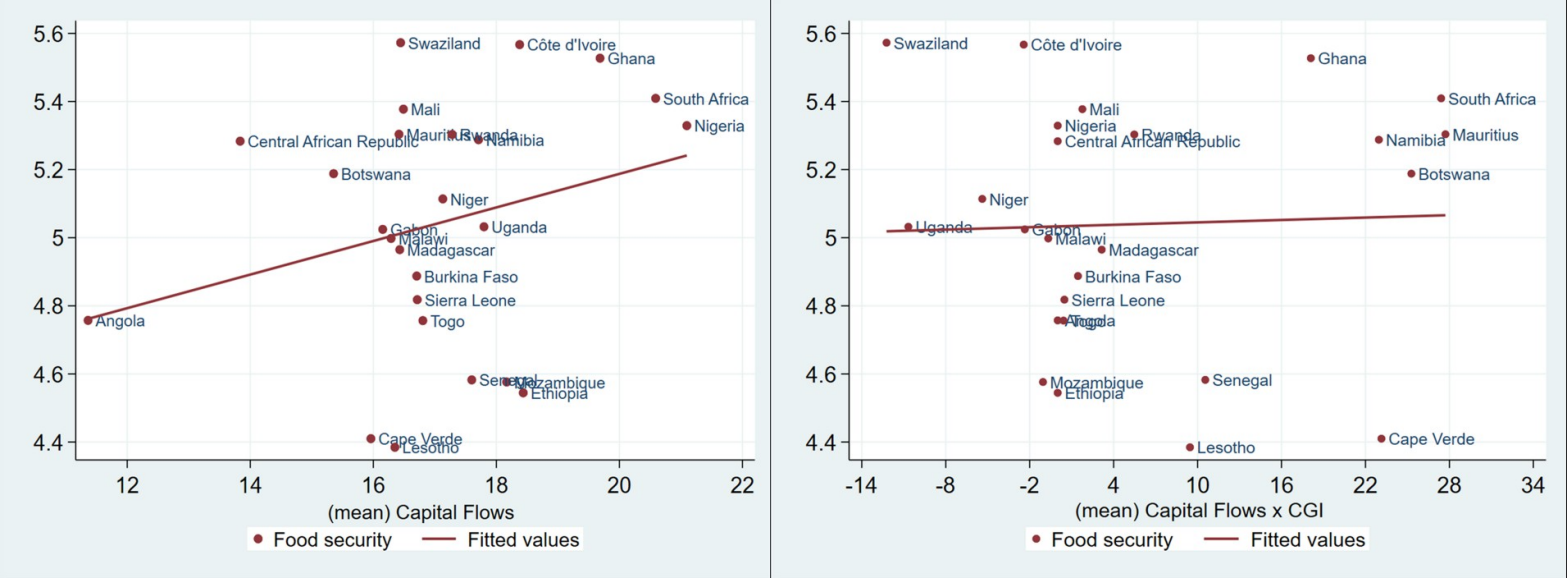
Food security, capital flows and the interaction of governance and capital flows in SSA (1996 to 2018). Source: Authors’ analysis from FAOSTAT, WGI and WDI.

**Fig 2:**
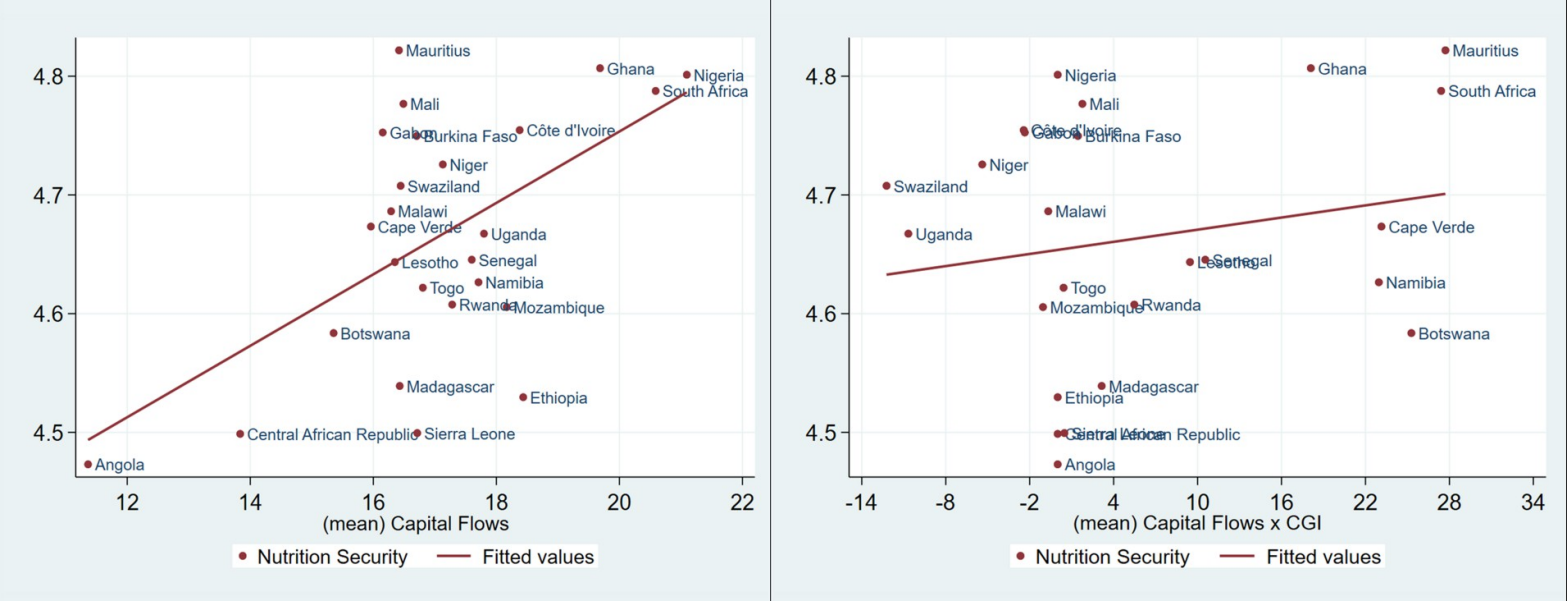
Nutrition security, capital flows and the interaction of governance and capital flows in SSA (1996 to 2018). Source: Authors’ analysis from FAOSTAT, WGI and WDI.

**Fig 3:**
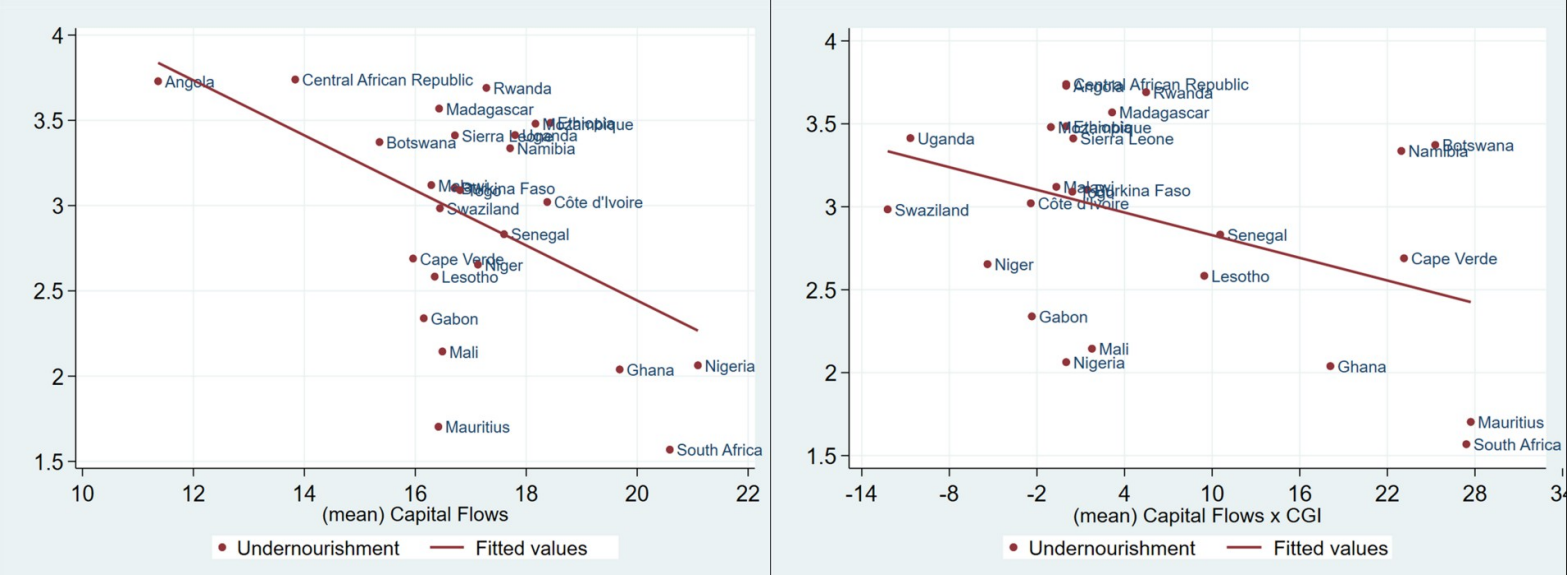
Undernourishment, capital flows and the interaction of governance and capital flows in SSA (1996 to 2018). Source: Authors’ analysis from FAOSTAT, WGI and WDI.

Fig 1 shows a positive linear relationship between food security and capital flows, and no relationship between capital flows-governance interaction and food security. Similarly, in Fig 2, a strong positive linear relationship is observed between nutrition security and capital flows, but such a relationship is weak between nutrition security and capital flows-governance interaction. On the other hand, the relationship between undernourishment and capital flows, and undernourishment and capital flows-governance interaction show a strong linear negative relationship as presented in Fig 3.

These preliminary results seem to suggest that countries with high capital flows may be more food and nutrition secure relatively to countries with less foreign capital inflows as defined in this study. Also, poor governance in the region may undermine the effect of capital flows on the food and nutrition security. These preliminary findings seem to suggest that even with limited inflows of foreign capital good governance may produce desirable results in some countries.

### 4.2. Impact of governance and capital flows on food and nutrition security

In this section we report and discuss the empirical findings from the GMM estimation of Eq 2. In Table 2 we present the difference-GMM results (Models 1–6) and the system-GMM results (Models 7–12) of the determinants of food and nutrition security. Panels A, B, C, D, and E report estimates for *aggregate capital flows*, *FDI*, *PE*, *ODA*, and *Remittances*, respectively. Full results are also reported in Tables D-F in the S1 Appendix.

**Table 2:** The impact of governance and capital flows on food and nutrition security and undernourishment.

	(1)	(2)	(3)	(4)	(5)	(6)	(7)	(8)	(9)	(10)	(11)	(12)
	Difference GMM						System GMM					
	Average Value of Food Production		Average Dietary Energy Supply Adequacy		Undernourishment		Average Value of Food Production		Average Dietary Energy Supply Adequacy		Undernourishment	
***Panel A***												
*Capital flows*	0.021		0.028**		-0.002**		0.002**		0.024**		-0.003***	
	(0.013)		(0.011)		(0.0001)		(0.000)		(0.005)		(0.001)	
CGI	0.008		0.005**		-0.049***		0.003		0.006***		-0.061***	
	(0.006)		(0.002)		(0.015)		(0.005)		(0.002)		(0.013)	
*Capital flows × CGI*		0.006		0.042**		-0.021**		0.007		0.062***		-0.033**
		(0.005)		(0.021)		(0.010)		(0.009)		(0.023)		(0.015)
*Lagged dep*. *Variable(t-1)*	0.751***	0.773***	0.923***	0.935***	0.900***	0.896***	0.889***	0.903***	1.029***	1.036***	0.917***	0.903***
	(0.025)	(0.024)	(0.017)	(0.017)	(0.032)	(0.032)	(0.017)	(0.017)	(0.014)	(0.014)	(0.022)	(0.021)
AR (1) *p-values*	0.000***	0.000***	0.001***	0.001***	0.000***	0.000***	0.000***	0.000***	0.001***	0.001***	0.000***	0.000***
AR (2) *p-values*	0.414	0.247	0.610	0.442	0.224	0.369	0.594	0.634	0.166	0.475	0.594	0.634
Hansen *p-values*	0.610	0.615	0.588	0.525	0.611	0.515	0.577	0.559	0.557	0.547	0.577	0.559
***Panel B***												
*FDI*	0.000		0.030***		-0.001		-0.001*		0.034***		-0.001*	
	(0.000)		(0.010)		(0.001)		(0.001)		(0.012)		(0.001)	
CGI	0.007		0.006***		-0.047***		0.003		0.005**		-0.061***	
	(0.006)		(0.002)		(0.015)		(0.005)		(0.002)		(0.013)	
*FDI × CGI*		0.000		0.060***		-0.001*		0.000		0.068*		-0.002***
		(0.000)		(0.009)		(0.001)		(0.000)		(0.035)		(0.001)
*Lagged dep*. *Variable(t-1)*	0.772***	0.775***	0.936***	0.935***	0.898***	0.894***	0.908***	0.904***	1.033***	1.032***	0.911***	0.904***
	(0.025)	(0.024)	(0.017)	(0.017)	(0.032)	(0.032)	(0.017)	(0.017)	(0.013)	(0.014)	(0.021)	(0.021)
AR (1) *p-values*	0.002***	0.003***	0.001***	0.001***	0.000***	0.000***	0.004***	0.005***	0.001***	0.002***	0.000***	0.001***
AR (2) *p-values*	0.556	0.496	0.551	0.494	0.548	0.460	0.455	0.547	0.381	0.458	0.578	0.574
Hansen *p-values*	0.546	0.426	0.608	0.556	0.519	0.520	0.568	0.570	0.624	0.607	0.600	0.578
***Panel C***												
*PE*	0.002		0.018		0.001		0.002		0.026*		-0.001	
	(0.001)		(0.013)		(0.001)		(0.001)		(0.016)		(0.001)	
CGI	0.008		0.006***		-0.050***		0.004		0.005**		-0.062***	
	(0.006)		(0.002)		(0.015)		(0.005)		(0.002)		(0.013)	
*PE× CGI*		0.000		0.000		-0.000		0.000		0.000*		-0.000
		(0.000)		(0.001)		(0.001)		(0.000)		(0.000)		(0.000)
*Lagged dep*. *Variable(t-1)*	0.758***	0.772***	0.927***	0.940***	0.900***	0.892***	0.887***	0.902***	1.034***	1.045***	0.917***	0.898***
	(0.025)	(0.024)	(0.017)	(0.017)	(0.032)	(0.032)	(0.017)	(0.017)	(0.014)	(0.013)	(0.021)	(0.021)
AR (1) *p-values*	0.004***	0.005***	0.002***	0.001***	0.000***	0.000***	0.005***	0.006***	0.003***	0.002***	0.000***	0.001***
AR (2) *p-values*	0.422	0.255	0.618	0.455	0.232	0.377	0.601	0.631	0.163	0.472	0.591	0.631
Hansen *p-values*	0.598	0.603	0.576	0.533	0.600	0.513	0.584	0.566	0.564	0.554	0.584	0.566
***Panel D***												
*ODA*	0.001***		0.002**		0.000		0.001***		0.025***		-0.002**	
	(0.000)		(0.001)		(0.003)		(0.000)		(0.002)		(0.001)	
CGI	0.008		0.006***		-0.048***		0.004		0.005**		-0.060***	
	(0.006)		(0.002)		(0.015)		(0.005)		(0.002)		(0.013)	
*ODA × CGI*		0.000		0.030***		-0.002***		0.000		0.050***		-0.003***
		(0.000)		(0.009)		(0.001)		(0.000)		(0.010)		(0.001)
*Lagged dep*. *Variable(t-1)*	0.773***	0.774***	0.938***	0.936***	0.900***	0.897***	0.901***	0.904***	1.033***	1.033***	0.913***	0.906***
	(0.025)	(0.024)	(0.017)	(0.017)	(0.032)	(0.032)	(0.017)	(0.017)	(0.014)	(0.014)	(0.022)	(0.021)
AR (1) *p-values*	0.006***	0.005***	0.002***	0.001***	0.001***	0.001***	0.004***	0.003***	0.001***	0.001***	0.001***	0.001***
AR (2) *p-values*	0.449	0.282	0.645	0.477	0.259	0.404	0.629	0.669	0.201	0.515	0.629	0.669
Hansen *p-values*	0.630	0.635	0.608	0.545	0.631	0.535	0.597	0.579	0.577	0.567	0.597	0.579
***Panel E***												
*Remittances*	0.001		0.000		-0.002**		0.001		0.002***		-0.005***	
	(0.001)		(0.000)		(0.000)		(0.001)		(0.000)		(0.002)	
CGI	0.006		0.006***		-0.048***		0.003		0.004**		-0.065***	
	(0.006)		(0.002)		(0.015)		(0.005)		(0.002)		(0.013)	
*Remittances × CGI*		0.000		0.001**		-0.002***		0.000		0.002**		-0.003***
		(0.000)		(0.000)		(0.001)		(0.000)		(0.000)		(0.001)
*Lagged dep*. *Variable(t-1)*	0.771***	0.775***	0.935***	0.940***	0.900***	0.903***	0.903***	0.905***	1.026***	1.036***	0.907***	0.910***
	(0.025)	(0.024)	(0.017)	(0.017)	(0.032)	(0.033)	(0.017)	(0.017)	(0.014)	(0.013)	(0.021)	(0.021)
AR (1) *p-values*	0.003***	0.003***	0.001***	0.001***	0.000***	0.000***	0.003***	0.004***	0.001***	0.001***	0.000***	0.000***
AR (2) *p-values*	0.437	0.270	0.633	0.465	0.247	0.392	0.617	0.657	0.189	0.498	0.617	0.657
Hansen *p-values*	0.642	0.647	0.620	0.557	0.643	0.547	0.609	0.591	0.589	0.579	0.609	0.591

*Source*: Authors. Data sources and definitions for all variables are provided in Table 1.

*Note*: Full models on Tables D-F in the S1 Appendix. All models included control variables, time fixed effect, and country fixed effect. All variables are in logarithmic form. Values in parentheses are standard errors of the estimates.

*** p < 0.01

** p < 0.05

* p < 0.1.

We follow Roodman (2009) [93] to address the problem of proliferation by using lags of endogenous variables (including capital flows, governance quality indicators, population growth, secondary school enrolments and the interactive capital flows x CGI term).

By estimating and reporting the two GMM estimates (Difference-GMM and System-GMM), we are able to test the model efficiency in addition to using undernourishment as an additional measure of food and nutritional security as robustness checks. More importantly, we conduct the misspecification diagnostics of the results using the Arellano-Bond statistics, AR (1) and AR (2), to test for the autocorrelation of the residuals. The test results reject the null hypothesis of no first-order residual serial correlation and accept the hypothesis of no second-order serial correlation. In addition, the Hansen test fails to reject the hypothesis of jointly valid instruments for all the estimated models, and the test statistic of overidentifying restrictions is insignificant, which suggests that the set of instruments employed fulfils the exogeneity condition required to obtained consistent estimates in the estimated models.

#### 4.2.1. Impact of governance and capital flows on food security

In Table 2, Models 1 and 2 report the results of the difference-GMM estimator and Models 7 and 8 report those of the system-GMM estimator. The full results are reported in Table D1 and D2 in the S1 Appendix. The results show that only ODA has a significant effect on food security, something not captured in the aggregate capital flows variable in the difference-GMM model. However, both ODA and aggregate capital flows are statistically significant in the system-GMM model, including FDI but with a negative effect. These results confirm results from previous studies [28, 34]. Few of the indicators of the composite governance index are significant, such as control of corruption, which shows positive significance across the models, with voice and accountability showing a negative and significant effect only in the difference-GMM model. The study by Mehta and Jha [70] also showed that corruption increases with food insecurity, globally. By employing a system GMM approach for a panel data of 15 SSA countries, Anser et al. [71] also show that government effectiveness and control of corruption as proxies for good governance improve food security as measured by the Food Production index. Our finding suggests that the effect of governance on food security is weak enough to complement the impact of capital flows on food security in Africa.

The negative and significant effect of FDI on food security in the system-GMM model is rather unexpected. However, since international investments such as FDI are channeled to different sectors of the economy this may have varying implications for food security. In this context, Mihalache-O’Keef and Li [72] isolate the effect of different types of FDI inflow in 56 developing countries on food security (measured as daily per capita calorie). They found that primary-sector FDI has a reducing effect on food security, while manufacturing improves it. Hence, this seems to suggest that the effects of different types of FDI should be analyzed separately to understand the disparity of FDI impact on development outcomes. Earlier studies based on dependency theory also show that a country’s reliance on foreign capital may widen the income gap between the rich and the poor, even as growth increases within the country [73, 74]. Such an argument has been extended to suggest that FDI may reduce food security by disproportionally creating losses for the poor [75, 76].

Furthermore, other factors that determine food security are the share of agricultural GDP with positive effect, population growth with a negative effect, and secondary school enrollment (only in the system-GMM) with an increasing effect on food security. Inflation rate is not significant across all models. Higher level of literacy is shown to have significant increasing effect on food security [52, 77]. This is because education promotes increased productivity and income, and access to other essential factors required to increase food security [77]. The value of food production may increase with the share of agricultural GDP; however, this may not translate into food security due to lack of infrastructure resulting in poor market linkages and high post-harvest losses [78]. Rising population has reducing effects on the quantity and quality of food available to people when the variations in the level of population growth is considered across most African countries.

#### 4.2.2. Impact of governance and capital flows on nutrition security

The difference-GMM results are reported in Table 2, Models 3 and 4, while the system-GMM estimates are reported in Models 9 and 10. Table E1 and E2 in the S1 Appendix contain the full results. Our results show that capital flows, FDI, ODA, governance index, and their interactions have a positive and strong effect on nutrition security. These results are more robust in the system-GMM models. The impact of governance on nutrition security becomes more obvious when endogeneity in the model is accounted for and when the variations in the level of the of governance are considered across countries in Africa. There is an indication that governance reinforces capital flows to enhance nutrition security in Africa. For example, Bain et al.’s [79] work on SSA countries indicates that a high level of corruption has a reducing effect on nutritional wellbeing in Africa. The effect of various interventions put in place to tackle the problem in the region has been minimal due to misappropriation of funds and the failure to accord the problem of nutrition security the attention it requires.

Among the element of governance indicators, political stability, governance effectiveness and control of corruption have a positive and significant effect on nutrition security, while rule of law has a reducing effect. This evidence is supported by a recent study that shows how weak institutions and bureaucratic corruption impact negatively on household food security in SSA [80]. Poor governance effectiveness also diminishes the performance of a given sector’s institutions and actors, as well as the concrete outcomes of policies [81]. Another study used panel data for 124 developing countries over the period 1984–2018 and results from a system GMM estimation seem to suggest that government stability, the rule of law, investment profile, and democratic accountability have positive impact on dietary energy supply [82]. Among our control variables, while inflation remains insignificant with a negative sign, agricultural GDP and secondary school enrollment have a positive growth effect on nutrition security. Studies in some countries in Africa have shown that parental education above primary school significantly improve child nutrition outcomes [39, 83]. Similarly, population growth has a negative effect on nutrition security.

#### 4.2.3. Impact of governance and capital flows on undernourishment

Turning to the impact on undernourishment, the difference-GMM results are reported in Models 5 and 6, while the system-GMM report are contained in Models 9 and 10 (Table 2). Table F1 and F2 in the S1 Appendix report the full results. Our results seem to suggest that aggregate capital flows, FDI, ODA, and remittances have a reducing effect on undernourishment, with even more robustness in the system-GMM model specifications. By using the Disability-Adjusted Life Years (DALYs) to measure global hunger burden, Gödecke et al. [84] show that economic growth is a major determinant of hunger reduction. In another study, Soriano and Garrido [85] use panel data for 27 developing countries to show that faster annual economic growth leads to more annual reduction in undernourishment. They pointed out that investments in health, education and access to drinking water are the enabling factors for reducing undernourishment in developing countries. A robust impact of capital flows on undernourishment may be felt when foreign direct investment and aid flows are channeled towards nutrition-sensitive programs. According to Mary et al. [86], a 10% increase in total nutrition-sensitive aid (food aid and emergency food aid) would reduce hunger by 1.1% on average in 2 years later. Their study suggests prioritization of specific nutrition-sensitive investments within the SDG agenda.

The components of governance that significantly reduce undernourishment include political stability, and control of corruption. Good governance quality is shown to reduce child undernutrition [87]. Other studies have confirmed that political stability in democracies is associated with a reduced burden of chronic and hidden hunger [84, 88]. The joint effects of the composite governance index and aggregate capital flows and their disaggregated components, including secondary school enrollment, have a reducing effect on undernourishment. Agricultural share of GDP and population growth have a positive and significant effect on undernourishment. Using undernourishment as a measure of food security, Pawlak and Kołodziejczak [49] showed that problems with maintaining food security are found with the greatest intensity in most African countries with a high share of agriculture in their GDP. This is due to adverse conditions hindering agricultural production and deficient infrastructure; thus, it may not have a significant effect on reducing undernutrition in SSA. The rise in SSA’s population means increase in the proportion of undernourished children in absolute numbers, thus making it difficult for SSA to reduce undernourishment to the barest minimum [89]. Bekana [90] shows that the quality of governance promotes innovations through its positive effect on human capital development, which is often measured by education enrolment. Parental education is a major contributor to nutritional status of children [39, 91], as it increases knowledge to make good nutritional choices and earning to purchase nutritious food. Results from the three indicators used in measuring food and nutrition outcomes show some variations, and this have important implications for policy to reduce hunger and malnutrition [92].

## 5. Concluding remarks

Recent years have witnessed an increasing interest in issues of food security in line also with the 2030 Agenda and the relevant Sustainable Development Goal 2 on zero hunger. At the same time, however, recent years have also witnessed a significant rise in the number of food insecure and malnourished people in the world, particularly in the SSA region. A few recent studies have also tried to examine the complex interaction between institutions, capital flows, and food and nutrition security. In this paper we delve deeper into the above nexus by estimating a series of dynamic panel data models to examine the impact of institutions and capital flows (in the form of ODA, FDI, Portfolio Equity and Remittances) on food security, nutrition security and undernourishment using panel data for 25 SSA countries over the period 1996 to 2018. One of the key contributions of the paper is the use of both aggregate and disaggregated capital flows to examine the impact on both food and nutrition security as well as on undernourishment, a dimension that has been surprisingly neglected in most of the relevant literature. We combine this with the interaction of various types of capital flows with an institutional quality index we constructed from various governance indicators and in order to examine also the impact of institutions on the overall nexus. We also employ a dynamic estimation methodology in the form of Difference-GMM and System-GMM estimators along with various misspecification diagnostics to deal with possible endogeneity issues. Our findings clearly demonstrate the importance of the heterogeneity approach and reflect on earlier work regarding the role of governance quality in the overall nexus between external capital flows and various measures of food and nutrition security which leads, and as expected, to an interesting variation in the results obtained, depending on the type of capital flows and the interaction with the governance indicators.

In the aftermath of the unprecedented Covid-19 pandemic it is imperative for the international development community to make a huge effort to intensify progress on the food & nutrition security front in the coming years and in order to compensate for the substantial progress made so far on this front, and which was undermined by the pandemic recently. We hope that the present study may help us to delve a little further on the important range of factors affecting food and nutrition security (and undernourishment) in the SSA region with some significant policy lessons emerging from our analysis, which include among others the centrality of quality of governance and its interaction with various types of capital flows in influencing final outcomes. And this seems to suggest that it is not so much the magnitude of the capital flows but their interaction with the levels of governance quality which needs to attract the particular attention of policy makers in order to enhance further food and nutrition security in the region.

## Supporting information

S1 Appendix (DOCX)
