# Supplementary material for: The combined effect of institutional quality and capital flows on food and nutrition security and undernourishment in Sub-Saharan Africa
Source: PLoS One. 2022 Oct 10;17(10):e0275345. doi: 10.1371/journal.pone.0275345 (PMC9550045; doi:10.1371/journal.pone.0275345)
Supplement: S1 Appendix — (DOCX) [file pone.0275345.s001.docx]

**Appendix**

## **Results of the correlation matrix of variables**

**Table A1: Correlation matrix in the first model of food security (average value of food production) determinants**

|  | ln_avg~p | ln_vae~i | ln_pve~i | ln_gee~i | ln_rqe~i | ln_rle~i | ln_cce~i | ln_fdi | ln_oda | ln_pef | ln_remit | ln_inf~n | ln_agr~p | ln_se_~r | ln_sp_~w |
| --- | --- | --- | --- | --- | --- | --- | --- | --- | --- | --- | --- | --- | --- | --- | --- |
| ln_avg_val~p | 1.0000 |  |  |  |  |  |  |  |  |  |  |  |  |  |  |
| ln_vae | -0.0288 | 1.0000 |  |  |  |  |  |  |  |  |  |  |  |  |  |
| ln_pve | -0.0086 | 0.2194 | 1.0000 |  |  |  |  |  |  |  |  |  |  |  |  |
| ln_gee | -0.0270 | 0.3045 | 0.2361 | 1.0000 |  |  |  |  |  |  |  |  |  |  |  |
| ln_rqe | -0.0459 | 0.2765 | 0.1525 | 0.2387 | 1.0000 |  |  |  |  |  |  |  |  |  |  |
| ln_rle | 0.0469 | 0.4330 | 0.3406 | 0.3467 | 0.4147 | 1.0000 |  |  |  |  |  |  |  |  |  |
| ln_cce | 0.1526 | 0.3712 | 0.3797 | 0.2726 | 0.3130 | 0.2572 | 1.0000 |  |  |  |  |  |  |  |  |
| ln_fdi | 0.0178 | -0.0286 | 0.1263 | -0.0805 | -0.0189 | 0.0277 | 0.1212 | 1.0000 |  |  |  |  |  |  |  |
| ln_oda | -0.1034 | -0.0693 | -0.2598 | -0.1675 | -0.1712 | -0.1653 | -0.2697 | 0.1900 | 1.0000 |  |  |  |  |  |  |
| ln_pef | 0.1648 | -0.0417 | -0.0686 | -0.0173 | -0.0639 | -0.0295 | 0.0360 | 0.2074 | 0.0435 | 1.0000 |  |  |  |  |  |
| ln_remit | -0.0715 | 0.0460 | -0.0557 | -0.1927 | -0.1919 | -0.1604 | 0.2022 | 0.2840 | 0.2067 | 0.2875 | 1.0000 |  |  |  |  |
| ln_inflation | -0.0598 | 0.1340 | 0.0081 | -0.0162 | 0.0713 | 0.2214 | -0.1490 | 0.0509 | 0.1725 | -0.0437 | 0.0796 | 1.0000 |  |  |  |
| ln_agricgdp | -0.1293 | -0.2409 | -0.3623 | -0.2143 | -0.3076 | -0.4177 | -0.5113 | 0.1199 | 0.4591 | -0.0123 | -0.0045 | -0.0052 | 1.0000 |  |  |
| ln_se_sec_~r | 0.0330 | 0.2567 | 0.3643 | 0.2224 | 0.1645 | 0.3074 | 0.5800 | 0.0916 | -0.2977 | 0.0563 | 0.4033 | 0.0572 | -0.4905 | 1.0000 |  |
| ln_sp_pop_~w | -0.2336 | -0.3868 | -0.3677 | -0.3432 | -0.3093 | -0.3579 | -0.4766 | 0.0248 | 0.3643 | -0.0330 | 0.1550 | 0.0505 | 0.3990 | -0.4969 | 1.0000 |

**Table A2: Correlation matrix in the second model of food security (average value of food production) determinants**

|  | ln_avg~p | ln_cgi | ln_fdi | ln_oda | ln_pef | ln_remit | ln_inf~n | ln_agr~p | ln_se_~r | ln_sp_~w |
| --- | --- | --- | --- | --- | --- | --- | --- | --- | --- | --- |
| ln_avg_val~p | 1.0000 |  |  |  |  |  |  |  |  |  |
| ln_cgi | 0.1065 | 1.0000 |  |  |  |  |  |  |  |  |
| ln_fdi | 0.0178 | 0.1478 | 1.0000 |  |  |  |  |  |  |  |
| ln_oda | -0.1034 | -0.2922 | 0.1900 | 1.0000 |  |  |  |  |  |  |
| ln_pef | 0.1648 | -0.0041 | 0.2074 | 0.0435 | 1.0000 |  |  |  |  |  |
| ln_remit | -0.0715 | 0.0760 | 0.2840 | 0.2067 | 0.2875 | 1.0000 |  |  |  |  |
| ln_inflation | -0.0598 | -0.0956 | 0.0509 | 0.1725 | -0.0437 | 0.0796 | 1.0000 |  |  |  |
| ln_agricgdp | -0.1293 | -0.4929 | 0.1199 | 0.4591 | -0.0123 | -0.0045 | -0.0052 | 1.0000 |  |  |
| ln_se_sec_~r | 0.0330 | 0.4932 | 0.0916 | -0.2977 | 0.0563 | 0.4033 | 0.0572 | -0.4905 | 1.0000 |  |
| ln_sp_pop_~w | -0.2336 | -0.4339 | 0.0248 | 0.3643 | -0.0330 | 0.1550 | 0.0505 | 0.3990 | -0.4969 | 1.0000 |

**Table A3: Correlation matrix in the third model of food security (average value of food production) determinants**

|  | ln_avg~p | ln_cgi~i | ln_cgi~a | ln_cg~ef | ln_cgi~t | ln_inf~n | ln_agr~p | ln_se_~r | ln_sp_~w |
| --- | --- | --- | --- | --- | --- | --- | --- | --- | --- |
| ln_avg_val~p | 1.0000 |  |  |  |  |  |  |  |  |
| ln_cgi_fdi | 0.1460 | 1.0000 |  |  |  |  |  |  |  |
| ln_cgi_oda | 0.1429 | 0.8677 | 1.0000 |  |  |  |  |  |  |
| ln_cgi_pef | 0.1146 | 0.3551 | 0.3494 | 1.0000 |  |  |  |  |  |
| ln_cgi_remit | 0.1346 | 0.8723 | 0.9769 | 0.3352 | 1.0000 |  |  |  |  |
| ln_inflation | -0.0598 | 0.0810 | 0.0271 | -0.0206 | 0.0263 | 1.0000 |  |  |  |
| ln_agricgdp | -0.1293 | -0.5582 | -0.4728 | -0.3328 | -0.4915 | -0.0052 | 1.0000 |  |  |
| ln_se_sec_~r | 0.0330 | 0.4979 | 0.5115 | 0.3022 | 0.5393 | 0.0572 | -0.4905 | 1.0000 |  |
| ln_sp_pop_~w | -0.2336 | -0.3348 | -0.3686 | -0.2961 | -0.3739 | 0.0505 | 0.3990 | -0.4969 | 1.0000 |

**Table A4: Correlation matrix in the fourth model of food security (average value of food production) determinants**

|  | ln_avg~p | ln_cgi | ln_cf | ln_inf~n | ln_agr~p | ln_se_~r | ln_sp_~w |
| --- | --- | --- | --- | --- | --- | --- | --- |
| ln_avg_val~p | 1.0000 |  |  |  |  |  |  |
| ln_cgi | 0.1065 | 1.0000 |  |  |  |  |  |
| ln_cf | 0.0552 | 0.0359 | 1.0000 |  |  |  |  |
| ln_inflation | -0.0598 | -0.0956 | 0.0515 | 1.0000 |  |  |  |
| ln_agricgdp | -0.1293 | -0.4929 | 0.1005 | -0.0052 | 1.0000 |  |  |
| ln_se_sec_~r | 0.0330 | 0.4932 | 0.1887 | 0.0572 | -0.4905 | 1.0000 |  |
| ln_sp_pop_~w | -0.2336 | -0.4339 | 0.1094 | 0.0505 | 0.3990 | -0.4969 | 1.0000 |

**Table A5: Correlation matrix in the fifth model of food security (average value of food production) determinants**

| ln_avg~p | ln_cg~cf | ln_inf~n | ln_agr~p | ln_se_~r | ln_sp_~w |  |
| --- | --- | --- | --- | --- | --- | --- |
| ln_avg_val~p | 1.0000 |  |  |  |  |  |
| ln_cgi_cf | 0.0982 | 1.0000 |  |  |  |  |
| ln_inflation | -0.0598 | -0.0827 | 1.0000 |  |  |  |
| ln_agricgdp | -0.1293 | -0.4943 | -0.0052 | 1.0000 |  |  |
| ln_se_sec_~r | 0.0330 | 0.4473 | 0.0572 | -0.4905 | 1.0000 |  |
| ln_sp_pop_~w | -0.2336 | -0.4107 | 0.0505 | 0.3990 | -0.4969 | 1.0000 |

**Table B1: Correlation matrix in the first model of nutrition security (dietary supply adequacy) determinants**

|  | ln_avg~a | ln_vae~i | ln_pve~i | ln_gee~i | ln_rqe~i | ln_rle~i | ln_cce~i | ln_fdi | ln_oda | ln_pef | ln_remit | ln_inf~n | ln_agr~p | ln_se_~r | ln_sp_~w |
| --- | --- | --- | --- | --- | --- | --- | --- | --- | --- | --- | --- | --- | --- | --- | --- |
| ln_avg_desa | 1.0000 |  |  |  |  |  |  |  |  |  |  |  |  |  |  |
| ln_vae | -0.0101 | 1.0000 |  |  |  |  |  |  |  |  |  |  |  |  |  |
| ln_pve | 0.1259 | 0.2194 | 1.0000 |  |  |  |  |  |  |  |  |  |  |  |  |
| ln_gee | -0.0977 | 0.3045 | 0.2361 | 1.0000 |  |  |  |  |  |  |  |  |  |  |  |
| ln_rqe | -0.0341 | 0.2765 | 0.1525 | 0.2387 | 1.0000 |  |  |  |  |  |  |  |  |  |  |
| ln_rle | 0.1178 | 0.4330 | 0.3406 | 0.3467 | 0.4147 | 1.0000 |  |  |  |  |  |  |  |  |  |
| ln_cce | 0.2179 | 0.3712 | 0.3797 | 0.2726 | 0.3130 | 0.2572 | 1.0000 |  |  |  |  |  |  |  |  |
| ln_fdi | 0.2567 | -0.0286 | 0.1263 | -0.0805 | -0.0189 | 0.0277 | 0.1212 | 1.0000 |  |  |  |  |  |  |  |
| ln_oda | -0.0591 | -0.0693 | -0.2598 | -0.1675 | -0.1712 | -0.1653 | -0.2697 | 0.1900 | 1.0000 |  |  |  |  |  |  |
| ln_pef | 0.3101 | -0.0417 | -0.0686 | -0.0173 | -0.0639 | -0.0295 | 0.0360 | 0.2074 | 0.0435 | 1.0000 |  |  |  |  |  |
| ln_remit | 0.5328 | 0.0460 | -0.0557 | -0.1927 | -0.1919 | -0.1604 | 0.2022 | 0.2840 | 0.2067 | 0.2875 | 1.0000 |  |  |  |  |
| ln_inflation | -0.0304 | 0.1340 | 0.0081 | -0.0162 | 0.0713 | 0.2214 | -0.1490 | 0.0509 | 0.1725 | -0.0437 | 0.0796 | 1.0000 |  |  |  |
| ln_agricgdp | -0.1090 | -0.2409 | -0.3623 | -0.2143 | -0.3076 | -0.4177 | -0.5113 | 0.1199 | 0.4591 | -0.0123 | -0.0045 | -0.0052 | 1.0000 |  |  |
| ln_se_sec_~r | 0.4064 | 0.2567 | 0.3643 | 0.2224 | 0.1645 | 0.3074 | 0.5800 | 0.0916 | -0.2977 | 0.0563 | 0.4033 | 0.0572 | -0.4905 | 1.0000 |  |
| ln_sp_pop_~w | -0.1856 | -0.3868 | -0.3677 | -0.3432 | -0.3093 | -0.3579 | -0.4766 | 0.0248 | 0.3643 | -0.0330 | 0.1550 | 0.0505 | 0.3990 | -0.4969 | 1.0000 |

**Table B2: Correlation matrix in the second model of nutrition security (dietary supply adequacy) determinants**

|  | ln_avg~a | ln_cgi | ln_fdi | ln_oda | ln_pef | ln_remit | ln_inf~n | ln_agr~p | ln_se_~r | ln_sp_~w |
| --- | --- | --- | --- | --- | --- | --- | --- | --- | --- | --- |
| ln_avg_desa | 1.0000 |  |  |  |  |  |  |  |  |  |
| ln_cgi | 0.2483 | 1.0000 |  |  |  |  |  |  |  |  |
| ln_fdi | 0.2567 | 0.1478 | 1.0000 |  |  |  |  |  |  |  |
| ln_oda | -0.0591 | -0.2922 | 0.1900 | 1.0000 |  |  |  |  |  |  |
| ln_pef | 0.3101 | -0.0041 | 0.2074 | 0.0435 | 1.0000 |  |  |  |  |  |
| ln_remit | 0.5328 | 0.0760 | 0.2840 | 0.2067 | 0.2875 | 1.0000 |  |  |  |  |
| ln_inflation | -0.0304 | -0.0956 | 0.0509 | 0.1725 | -0.0437 | 0.0796 | 1.0000 |  |  |  |
| ln_agricgdp | -0.1090 | -0.4929 | 0.1199 | 0.4591 | -0.0123 | -0.0045 | -0.0052 | 1.0000 |  |  |
| ln_se_sec_~r | 0.4064 | 0.4932 | 0.0916 | -0.2977 | 0.0563 | 0.4033 | 0.0572 | -0.4905 | 1.0000 |  |
| ln_sp_pop_~w | -0.1856 | -0.4339 | 0.0248 | 0.3643 | -0.0330 | 0.1550 | 0.0505 | 0.3990 | -0.4969 | 1.0000 |

**Table B3: Correlation matrix in the third model of nutrition security (dietary supply adequacy) determinants**

|  | ln_avg~a | ln_cgi~i | ln_cgi~a | ln_cg~ef | ln_cgi~t | ln_inf~n | ln_agr~p | ln_se_~r | ln_sp_~w |
| --- | --- | --- | --- | --- | --- | --- | --- | --- | --- |
| ln_avg_desa | 1.0000 |  |  |  |  |  |  |  |  |
| ln_cgi_fdi | 0.1968 | 1.0000 |  |  |  |  |  |  |  |
| ln_cgi_oda | 0.2672 | 0.8677 | 1.0000 |  |  |  |  |  |  |
| ln_cgi_pef | 0.2362 | 0.3551 | 0.3494 | 1.0000 |  |  |  |  |  |
| ln_cgi_remit | 0.2668 | 0.8723 | 0.9769 | 0.3352 | 1.0000 |  |  |  |  |
| ln_inflation | -0.0304 | 0.0810 | 0.0271 | -0.0206 | 0.0263 | 1.0000 |  |  |  |
| ln_agricgdp | -0.1090 | -0.5582 | -0.4728 | -0.3328 | -0.4915 | -0.0052 | 1.0000 |  |  |
| ln_se_sec_~r | 0.4064 | 0.4979 | 0.5115 | 0.3022 | 0.5393 | 0.0572 | -0.4905 | 1.0000 |  |
| ln_sp_pop_~w | -0.1856 | -0.3348 | -0.3686 | -0.2961 | -0.3739 | 0.0505 | 0.3990 | -0.4969 | 1.0000 |

**Table B4: Correlation matrix in the fourth model of nutrition security (dietary supply adequacy) determinants**

|  | ln_avg~a | ln_cgi | ln_cf | ln_inf~n | ln_agr~p | ln_se_~r | ln_sp_~w |
| --- | --- | --- | --- | --- | --- | --- | --- |
| ln_avg_desa | 1.0000 |  |  |  |  |  |  |
| ln_cgi | 0.2483 | 1.0000 |  |  |  |  |  |
| ln_cf | 0.4810 | 0.0359 | 1.0000 |  |  |  |  |
| ln_inflation | -0.0304 | -0.0956 | 0.0515 | 1.0000 |  |  |  |
| ln_agricgdp | -0.1090 | -0.4929 | 0.1005 | -0.0052 | 1.0000 |  |  |
| ln_se_sec_~r | 0.4064 | 0.4932 | 0.1887 | 0.0572 | -0.4905 | 1.0000 |  |
| ln_sp_pop_~w | -0.1856 | -0.4339 | 0.1094 | 0.0505 | 0.3990 | -0.4969 | 1.0000 |

**Table B5: Correlation matrix in the fifth model of nutrition security (dietary supply adequacy) determinants**

|  | ln_avg~a | ln_cg~cf | ln_inf~n | ln_agr~p | ln_se_~r | ln_sp_~w |
| --- | --- | --- | --- | --- | --- | --- |
| ln_avg_desa | 1.0000 |  |  |  |  |  |
| ln_cgi_cf | 0.1945 | 1.0000 |  |  |  |  |
| ln_inflation | -0.0304 | -0.0827 | 1.0000 |  |  |  |
| ln_agricgdp | -0.1090 | -0.4943 | -0.0052 | 1.0000 |  |  |
| ln_se_sec_~r | 0.4064 | 0.4473 | 0.0572 | -0.4905 | 1.0000 |  |
| ln_sp_pop_~w | -0.1856 | -0.4107 | 0.0505 | 0.3990 | -0.4969 | 1.0000 |

**Table C1: Correlation matrix in the first model of hunger (undernourishment) determinants**

|  | ln_und~r | ln_vae~i | ln_pve~i | ln_gee~i | ln_rqe~i | ln_rle~i | ln_cce~i | ln_fdi | ln_oda | ln_pef | ln_remit | ln_inf~n | ln_agr~p | ln_se_~r | ln_sp_~w |
| --- | --- | --- | --- | --- | --- | --- | --- | --- | --- | --- | --- | --- | --- | --- | --- |
| ln_undernour | 1.0000 |  |  |  |  |  |  |  |  |  |  |  |  |  |  |
| ln_vae | -0.0489 | 1.0000 |  |  |  |  |  |  |  |  |  |  |  |  |  |
| ln_pve | -0.2915 | 0.2194 | 1.0000 |  |  |  |  |  |  |  |  |  |  |  |  |
| ln_gee | 0.0424 | 0.3045 | 0.2361 | 1.0000 |  |  |  |  |  |  |  |  |  |  |  |
| ln_rqe | -0.0362 | 0.2765 | 0.1525 | 0.2387 | 1.0000 |  |  |  |  |  |  |  |  |  |  |
| ln_rle | -0.2049 | 0.4330 | 0.3406 | 0.3467 | 0.4147 | 1.0000 |  |  |  |  |  |  |  |  |  |
| ln_cce | -0.3097 | 0.3712 | 0.3797 | 0.2726 | 0.3130 | 0.2572 | 1.0000 |  |  |  |  |  |  |  |  |
| ln_fdi | -0.2336 | -0.0286 | 0.1263 | -0.0805 | -0.0189 | 0.0277 | 0.1212 | 1.0000 |  |  |  |  |  |  |  |
| ln_oda | 0.1338 | -0.0693 | -0.2598 | -0.1675 | -0.1712 | -0.1653 | -0.2697 | 0.1900 | 1.0000 |  |  |  |  |  |  |
| ln_pef | -0.2197 | -0.0417 | -0.0686 | -0.0173 | -0.0639 | -0.0295 | 0.0360 | 0.2074 | 0.0435 | 1.0000 |  |  |  |  |  |
| ln_remit | -0.4294 | 0.0460 | -0.0557 | -0.1927 | -0.1919 | -0.1604 | 0.2022 | 0.2840 | 0.2067 | 0.2875 | 1.0000 |  |  |  |  |
| ln_inflation | 0.0038 | 0.1340 | 0.0081 | -0.0162 | 0.0713 | 0.2214 | -0.1490 | 0.0509 | 0.1725 | -0.0437 | 0.0796 | 1.0000 |  |  |  |
| ln_agricgdp | 0.1977 | -0.2409 | -0.3623 | -0.2143 | -0.3076 | -0.4177 | -0.5113 | 0.1199 | 0.4591 | -0.0123 | -0.0045 | -0.0052 | 1.0000 |  |  |
| ln_se_sec_~r | -0.4572 | 0.2567 | 0.3643 | 0.2224 | 0.1645 | 0.3074 | 0.5800 | 0.0916 | -0.2977 | 0.0563 | 0.4033 | 0.0572 | -0.4905 | 1.0000 |  |
| ln_sp_pop_~w | 0.2392 | -0.3868 | -0.3677 | -0.3432 | -0.3093 | -0.3579 | -0.4766 | 0.0248 | 0.3643 | -0.0330 | 0.1550 | 0.0505 | 0.3990 | -0.4969 | 1.0000 |

**Table C2: Correlation matrix in the second model of hunger (undernourishment) determinants**

|  | ln_und~r | ln_cgi | ln_fdi | ln_oda | ln_pef | ln_remit | ln_inf~n | ln_agr~p | ln_se_~r | ln_sp_~w |
| --- | --- | --- | --- | --- | --- | --- | --- | --- | --- | --- |
| ln_undernour | 1.0000 |  |  |  |  |  |  |  |  |  |
| ln_cgi | -0.3090 | 1.0000 |  |  |  |  |  |  |  |  |
| ln_fdi | -0.2336 | 0.1478 | 1.0000 |  |  |  |  |  |  |  |
| ln_oda | 0.1338 | -0.2922 | 0.1900 | 1.0000 |  |  |  |  |  |  |
| ln_pef | -0.2197 | -0.0041 | 0.2074 | 0.0435 | 1.0000 |  |  |  |  |  |
| ln_remit | -0.4294 | 0.0760 | 0.2840 | 0.2067 | 0.2875 | 1.0000 |  |  |  |  |
| ln_inflation | 0.0038 | -0.0956 | 0.0509 | 0.1725 | -0.0437 | 0.0796 | 1.0000 |  |  |  |
| ln_agricgdp | 0.1977 | -0.4929 | 0.1199 | 0.4591 | -0.0123 | -0.0045 | -0.0052 | 1.0000 |  |  |
| ln_se_sec_~r | -0.4572 | 0.4932 | 0.0916 | -0.2977 | 0.0563 | 0.4033 | 0.0572 | -0.4905 | 1.0000 |  |
| ln_sp_pop_~w | 0.2392 | -0.4339 | 0.0248 | 0.3643 | -0.0330 | 0.1550 | 0.0505 | 0.3990 | -0.4969 | 1.0000 |

**Table C3: Correlation matrix in the third model of hunger (undernourishment) determinants**

| ln_und~r | ln_cgi~i | ln_cgi~a | ln_cg~ef | ln_cgi~t | ln_inf~n | ln_agr~p | ln_se_~r | ln_sp_~w |  |
| --- | --- | --- | --- | --- | --- | --- | --- | --- | --- |
| ln_undernour | 1.0000 |  |  |  |  |  |  |  |  |
| ln_cgi_fdi | -0.3056 | 1.0000 |  |  |  |  |  |  |  |
| ln_cgi_oda | -0.3848 | 0.8677 | 1.0000 |  |  |  |  |  |  |
| ln_cgi_pef | -0.2784 | 0.3551 | 0.3494 | 1.0000 |  |  |  |  |  |
| ln_cgi_remit | -0.3870 | 0.8723 | 0.9769 | 0.3352 | 1.0000 |  |  |  |  |
| ln_inflation | 0.0038 | 0.0810 | 0.0271 | -0.0206 | 0.0263 | 1.0000 |  |  |  |
| ln_agricgdp | 0.1977 | -0.5582 | -0.4728 | -0.3328 | -0.4915 | -0.0052 | 1.0000 |  |  |
| ln_se_sec_~r | -0.4572 | 0.4979 | 0.5115 | 0.3022 | 0.5393 | 0.0572 | -0.4905 | 1.0000 |  |
| ln_sp_pop_~w | 0.2392 | -0.3348 | -0.3686 | -0.2961 | -0.3739 | 0.0505 | 0.3990 | -0.4969 | 1.0000 |

**Table C4: Correlation matrix in the fourth model of hunger (undernourishment) determinants**

|  | ln_und~r | ln_cgi | ln_cf | ln_inf~n | ln_agr~p | ln_se_~r | ln_sp_~w |
| --- | --- | --- | --- | --- | --- | --- | --- |
| ln_undernour | 1.0000 |  |  |  |  |  |  |
| ln_cgi | -0.3090 | 1.0000 |  |  |  |  |  |
| ln_cf | -0.3664 | 0.0359 | 1.0000 |  |  |  |  |
| ln_inflation | 0.0038 | -0.0956 | 0.0515 | 1.0000 |  |  |  |
| ln_agricgdp | 0.1977 | -0.4929 | 0.1005 | -0.0052 | 1.0000 |  |  |
| ln_se_sec_~r | -0.4572 | 0.4932 | 0.1887 | 0.0572 | -0.4905 | 1.0000 |  |
| ln_sp_pop_~w | 0.2392 | -0.4339 | 0.1094 | 0.0505 | 0.3990 | -0.4969 | 1.0000 |

**Table C5: Correlation matrix in the fifth model of hunger (undernourishment) determinants**

|  | ln_und~r | ln_cg~cf | ln_inf~n | ln_agr~p | ln_se_~r | ln_sp_~w |
| --- | --- | --- | --- | --- | --- | --- |
| ln_undernour | 1.0000 |  |  |  |  |  |
| ln_cgi_cf | -0.2479 | 1.0000 |  |  |  |  |
| ln_inflation | 0.0038 | -0.0827 | 1.0000 |  |  |  |
| ln_agricgdp | 0.1977 | -0.4943 | -0.0052 | 1.0000 |  |  |
| ln_se_sec_~r | -0.4572 | 0.4473 | 0.0572 | -0.4905 | 1.0000 |  |
| ln_sp_pop_~w | 0.2392 | -0.4107 | 0.0505 | 0.3990 | -0.4969 | 1.0000 |

## **Full regression results**

**Table D1.** **The impact of governance and capital flows on average value of food production (Difference GMM estimates)**

| Dep. Variable: Average value of food production (AVFP) | (1) | (2) | (3) | (4) | (5) | (6) | (7) | (8) | (9) | (10) | (11) | (12) | (13) | (14) | (15) |
| --- | --- | --- | --- | --- | --- | --- | --- | --- | --- | --- | --- | --- | --- | --- | --- |
|  |  |  |  |  |  |  |  |  |  |  |  |  |  |  |  |
| Lagged dep. Variable(t-1) | 0.757*** | 0.744*** | 0.759*** | 0.758*** | 0.737*** | 0.772*** | 0.758*** | 0.773*** | 0.771*** | 0.751*** | 0.775*** | 0.772*** | 0.774*** | 0.775*** | 0.773*** |
|  | (0.025) | (0.025) | (0.025) | (0.025) | (0.026) | (0.025) | (0.025) | (0.025) | (0.025) | (0.025) | (0.024) | (0.024) | (0.024) | (0.024) | (0.024) |
| Voice and accountability score | -0.006* | -0.005* | -0.005* | -0.006* | -0.005* |  |  |  |  |  |  |  |  |  |  |
|  | (0.003) | (0.003) | (0.003) | (0.003) | (0.003) |  |  |  |  |  |  |  |  |  |  |
| Political stability score | 0.000 | 0.000 | 0.000 | 0.000 | -0.000 |  |  |  |  |  |  |  |  |  |  |
|  | (0.003) | (0.003) | (0.003) | (0.003) | (0.003) |  |  |  |  |  |  |  |  |  |  |
| Government effectiveness score | 0.001 | 0.001 | 0.001 | 0.001 | 0.001 |  |  |  |  |  |  |  |  |  |  |
|  | (0.002) | (0.002) | (0.002) | (0.002) | (0.002) |  |  |  |  |  |  |  |  |  |  |
| Regulatory quality score | 0.002 | 0.002 | 0.002 | 0.002 | 0.002 |  |  |  |  |  |  |  |  |  |  |
|  | (0.002) | (0.002) | (0.002) | (0.002) | (0.002) |  |  |  |  |  |  |  |  |  |  |
| Rule of law score | 0.000 | -0.000 | 0.000 | -0.000 | -0.000 |  |  |  |  |  |  |  |  |  |  |
|  | (0.003) | (0.003) | (0.003) | (0.003) | (0.003) |  |  |  |  |  |  |  |  |  |  |
| Control of corruption score | 0.050*** | 0.049*** | 0.048*** | 0.044** | 0.051*** |  |  |  |  |  |  |  |  |  |  |
|  | (0.019) | (0.019) | (0.019) | (0.018) | (0.019) |  |  |  |  |  |  |  |  |  |  |
| Composite governance index |  |  |  |  |  | 0.007 | 0.008 | 0.008 | 0.006 | 0.008 |  |  |  |  |  |
| (CGI) |  |  |  |  |  | (0.006) | (0.006) | (0.006) | (0.006) | (0.006) |  |  |  |  |  |
| Foreign Direct Investment (FDI) | 0.001 |  |  |  |  | 0.000 |  |  |  |  |  |  |  |  |  |
|  | (0.001) |  |  |  |  | (0.000) |  |  |  |  |  |  |  |  |  |
| Portfolio Equity (PE) |  | 0.002 |  |  |  |  | 0.002 |  |  |  |  |  |  |  |  |
|  |  | (0.001) |  |  |  |  | (0.001) |  |  |  |  |  |  |  |  |
| Official Development Assistance |  |  | 0.001*** |  |  |  |  | 0.001*** |  |  |  |  |  |  |  |
| (ODA) |  |  | (0.000) |  |  |  |  | (0.000) |  |  |  |  |  |  |  |
| Remittances |  |  |  | 0.001 |  |  |  |  | 0.001 |  |  |  |  |  |  |
|  |  |  |  | (0.001) |  |  |  |  | (0.001) |  |  |  |  |  |  |
| Capital flows (CF) |  |  |  |  | 0.019 |  |  |  |  | 0.021 |  |  |  |  |  |
|  |  |  |  |  | (0.011) |  |  |  |  | (0.013) |  |  |  |  |  |
| CGI x FDI |  |  |  |  |  |  |  |  |  |  | 0.000 |  |  |  |  |
|  |  |  |  |  |  |  |  |  |  |  | (0.000) |  |  |  |  |
| CGI x PE |  |  |  |  |  |  |  |  |  |  |  | 0.000 |  |  |  |
|  |  |  |  |  |  |  |  |  |  |  |  | (0.000) |  |  |  |
| CGI x ODA |  |  |  |  |  |  |  |  |  |  |  |  | 0.000 |  |  |
|  |  |  |  |  |  |  |  |  |  |  |  |  | (0.000) |  |  |
| CGI x Remittances |  |  |  |  |  |  |  |  |  |  |  |  |  | 0.000 |  |
|  |  |  |  |  |  |  |  |  |  |  |  |  |  | (0.000) |  |
| CGI x CF |  |  |  |  |  |  |  |  |  |  |  |  |  |  | 0.006 |
|  |  |  |  |  |  |  |  |  |  |  |  |  |  |  | (0.005) |
| Inflation | 0.000 | 0.001 | 0.000 | 0.000 | 0.001 | 0.001 | 0.001 | 0.001 | 0.001 | 0.001 | 0.001 | 0.001 | 0.001 | 0.001 | 0.001 |
|  | (0.002) | (0.002) | (0.002) | (0.002) | (0.002) | (0.002) | (0.002) | (0.002) | (0.002) | (0.002) | (0.002) | (0.002) | (0.002) | (0.002) | (0.002) |
| Share of agriculture in GDP | 0.018** | 0.018** | 0.018** | 0.018** | 0.019** | 0.018** | 0.018** | 0.019** | 0.019** | 0.019** | 0.018** | 0.017** | 0.018** | 0.018** | 0.018** |
|  | (0.008) | (0.008) | (0.008) | (0.008) | (0.008) | (0.008) | (0.008) | (0.008) | (0.008) | (0.008) | (0.008) | (0.008) | (0.008) | (0.008) | (0.008) |
| Secondary school enrolment | 0.010 | 0.012 | 0.007 | 0.006 | 0.008 | 0.012 | 0.014 | 0.009 | 0.007 | 0.010 | 0.011 | 0.010 | 0.011 | 0.011 | 0.012 |
|  | (0.009) | (0.009) | (0.010) | (0.010) | (0.009) | (0.009) | (0.009) | (0.009) | (0.010) | (0.009) | (0.009) | (0.009) | (0.009) | (0.009) | (0.009) |
| Population growth | -0.013*** | -0.015*** | -0.016*** | -0.015*** | -0.017*** | -0.017*** | -0.016*** | -0.017*** | -0.017*** | -0.017*** | -0.018*** | -0.017*** | -0.018*** | -0.017*** | -0.019*** |
|  | (0.003) | (0.003) | (0.003) | (0.003) | (0.003) | (0.003) | (0.003) | (0.003) | (0.003) | (0.003) | (0.003) | (0.003) | (0.003) | (0.003) | (0.003) |
| Constant | 1.152*** | 1.210*** | 1.129*** | 1.152*** | 1.217*** | 1.079*** | 1.135*** | 1.059*** | 1.082*** | 1.142*** | 1.075*** | 1.095*** | 1.077*** | 1.071*** | 1.081*** |
|  | (0.118) | (0.119) | (0.118) | (0.118) | (0.119) | (0.116) | (0.117) | (0.117) | (0.116) | (0.117) | (0.116) | (0.115) | (0.116) | (0.116) | (0.115) |
| Observations | 375 | 375 | 375 | 375 | 375 | 375 | 375 | 375 | 375 | 375 | 375 | 375 | 375 | 375 | 375 |
| Number of countries | 25 | 25 | 25 | 25 | 25 | 25 | 25 | 25 | 25 | 25 | 25 | 25 | 25 | 25 | 25 |
| Test (p-values) |  |  |  |  |  |  |  |  |  |  |  |  |  |  |  |
| AR (1) p-values | 0.002*** | 0.004*** | 0.005*** | 0.004*** | 0.001*** | 0.002*** | 0.004*** | 0.006*** | 0.003*** | 0.000*** | 0.003*** | 0.005*** | 0.005*** | 0.003*** | 0.000*** |
| AR (2) p-values | 0.556 | 0.43 | 0.449 | 0.437 | 0.414 | 0.556 | 0.422 | 0.449 | 0.437 | 0.414 | 0.496 | 0.255 | 0.282 | 0.270 | 0.247 |
| Harsen test p-values | 0.546 | 0.598 | 0.630 | 0.642 | 0.610 | 0.546 | 0.598 | 0.630 | 0.642 | 0.610 | 0.426 | 0.603 | 0.635 | 0.647 | 0.615 |

Note: Data sources and definitions for all variables are provided in Table 1

Standard errors in parentheses

*** p<0.01, ** p<0.05, * p<0.1



**Table D2: The impact of governance and capital flows on average value of food production (System GMM estimates)**

| Dep. Variable: Average value of food production (AVFP) | (1) | (2) | (3) | (4) | (5) | (6) | (7) | (8) | (9) | (10) | (11) | (12) | (13) | (14) | (15) |
| --- | --- | --- | --- | --- | --- | --- | --- | --- | --- | --- | --- | --- | --- | --- | --- |
| Lagged dep. Variable(t-1) | 0.904*** | 0.883*** | 0.897*** | 0.899*** | 0.885*** | 0.908*** | 0.887*** | 0.901*** | 0.903*** | 0.889*** | 0.904*** | 0.902*** | 0.904*** | 0.905*** | 0.903*** |
|  | (0.017) | (0.017) | (0.017) | (0.017) | (0.017) | (0.017) | (0.017) | (0.017) | (0.017) | (0.017) | (0.017) | (0.017) | (0.017) | (0.017) | (0.017) |
| Voice and accountability score | -0.001 | -0.001 | -0.001 | -0.001 | -0.004* |  |  |  |  |  |  |  |  |  |  |
|  | (0.003) | (0.003) | (0.003) | (0.003) | (0.003) |  |  |  |  |  |  |  |  |  |  |
| Political stability score | -0.000 | -0.001 | -0.001 | -0.001 | -0.001 |  |  |  |  |  |  |  |  |  |  |
|  | (0.003) | (0.003) | (0.003) | (0.003) | (0.003) |  |  |  |  |  |  |  |  |  |  |
| Government effectiveness score | 0.000 | 0.000 | 0.000 | 0.001 | 0.001 |  |  |  |  |  |  |  |  |  |  |
|  | (0.002) | (0.002) | (0.002) | (0.002) | (0.002) |  |  |  |  |  |  |  |  |  |  |
| Regulatory quality score | 0.002 | 0.002 | 0.002 | 0.002 | 0.002 |  |  |  |  |  |  |  |  |  |  |
|  | (0.002) | (0.002) | (0.002) | (0.002) | (0.002) |  |  |  |  |  |  |  |  |  |  |
| Rule of law score | -0.001 | -0.001 | -0.001 | -0.001 | -0.001 |  |  |  |  |  |  |  |  |  |  |
|  | (0.003) | (0.003) | (0.003) | (0.003) | (0.003) |  |  |  |  |  |  |  |  |  |  |
| Control of corruption score | 0.005 | 0.003 | 0.004 | 0.001 | 0.005** |  |  |  |  |  |  |  |  |  |  |
|  | (0.017) | (0.016) | (0.016) | (0.017) | (0.002) |  |  |  |  |  |  |  |  |  |  |
| Composite governance index (CGI) |  |  |  |  |  | 0.003 | 0.004 | 0.004 | 0.003 | 0.003 |  |  |  |  |  |
|  |  |  |  |  |  | (0.005) | (0.005) | (0.005) | (0.005) | (0.005) |  |  |  |  |  |
| Foreign Direct Investment (FDI) | -0.001* |  |  |  |  | -0.001* |  |  |  |  |  |  |  |  |  |
|  | (0.001) |  |  |  |  | (0.001) |  |  |  |  |  |  |  |  |  |
| Portfolio Equity (PE) |  | 0.002 |  |  |  |  | 0.002 |  |  |  |  |  |  |  |  |
|  |  | (0.001) |  |  |  |  | (0.001) |  |  |  |  |  |  |  |  |
| Official Development Assistance |  |  | 0.001*** |  |  |  |  | 0.001*** |  |  |  |  |  |  |  |
| (ODA) |  |  | (0.000) |  |  |  |  | (0.000) |  |  |  |  |  |  |  |
| Remittances |  |  |  | 0.001 |  |  |  |  | 0.001 |  |  |  |  |  |  |
|  |  |  |  | (0.001) |  |  |  |  | (0.001) |  |  |  |  |  |  |
| Capital flows (CF) |  |  |  |  | 0.030* |  |  |  |  | 0.002** |  |  |  |  |  |
|  |  |  |  |  | (0.018) |  |  |  |  | (0.000) |  |  |  |  |  |
| CGI x FDI |  |  |  |  |  |  |  |  |  |  | 0.000 |  |  |  |  |
|  |  |  |  |  |  |  |  |  |  |  | (0.000) |  |  |  |  |
| CGI x PE |  |  |  |  |  |  |  |  |  |  |  | 0.000 |  |  |  |
|  |  |  |  |  |  |  |  |  |  |  |  | (0.000) |  |  |  |
| CGI x ODA |  |  |  |  |  |  |  |  |  |  |  |  | 0.000 |  |  |
|  |  |  |  |  |  |  |  |  |  |  |  |  | (0.000) |  |  |
| CGI x Remittances |  |  |  |  |  |  |  |  |  |  |  |  |  | 0.000 |  |
|  |  |  |  |  |  |  |  |  |  |  |  |  |  | (0.000) |  |
| CGI x CF |  |  |  |  |  |  |  |  |  |  |  |  |  |  | 0.007 |
|  |  |  |  |  |  |  |  |  |  |  |  |  |  |  | (0.009) |
| Inflation | 0.000 | 0.001 | 0.000 | 0.001 | 0.001 | 0.000 | 0.001 | 0.000 | 0.001 | 0.001 | 0.000 | 0.000 | 0.001 | 0.001 | 0.001 |
|  | (0.002) | (0.002) | (0.002) | (0.002) | (0.002) | (0.002) | (0.002) | (0.002) | (0.002) | (0.002) | (0.002) | (0.002) | (0.002) | (0.002) | (0.002) |
| Share of agriculture in GDP | 0.021*** | 0.021*** | 0.021*** | 0.021*** | 0.021*** | 0.021*** | 0.022*** | 0.020*** | 0.021*** | 0.021*** | 0.021*** | 0.020*** | 0.021*** | 0.022*** | 0.021*** |
|  | (0.007) | (0.007) | (0.007) | (0.007) | (0.007) | (0.007) | (0.007) | (0.007) | (0.007) | (0.007) | (0.007) | (0.007) | (0.007) | (0.007) | (0.007) |
| Secondary school enrolment | 0.020** | 0.019** | 0.023*** | 0.022** | 0.022** | 0.020** | 0.019** | 0.023*** | 0.023*** | 0.022*** | 0.020** | 0.020** | 0.020** | 0.020** | 0.019** |
|  | (0.009) | (0.009) | (0.009) | (0.009) | (0.009) | (0.008) | (0.008) | (0.008) | (0.009) | (0.008) | (0.008) | (0.008) | (0.008) | (0.008) | (0.008) |
| Population growth | -0.058*** | -0.061*** | -0.053*** | -0.059*** | -0.059*** | -0.061*** | -0.065*** | -0.057*** | -0.063*** | -0.064*** | -0.061*** | -0.060*** | -0.061*** | -0.062*** | -0.061*** |
|  | (0.015) | (0.015) | (0.015) | (0.015) | (0.015) | (0.014) | (0.014) | (0.015) | (0.014) | (0.014) | (0.014) | (0.014) | (0.014) | (0.014) | (0.014) |
| Constant | 0.458*** | 0.528*** | 0.445*** | 0.462*** | 0.503*** | 0.434*** | 0.500*** | 0.422*** | 0.438*** | 0.476*** | 0.436*** | 0.452*** | 0.433*** | 0.426*** | 0.437*** |
|  | (0.083) | (0.084) | (0.083) | (0.082) | (0.083) | (0.083) | (0.085) | (0.084) | (0.083) | (0.084) | (0.082) | (0.080) | (0.082) | (0.082) | (0.081) |
| Observations | 400 | 400 | 400 | 400 | 400 | 400 | 400 | 400 | 400 | 400 | 400 | 400 | 400 | 400 | 400 |
| Number of countries | 25 | 25 | 25 | 25 | 25 | 25 | 25 | 25 | 25 | 25 | 25 | 25 | 25 | 25 | 25 |
| Test (p-values) |  |  |  |  |  |  |  |  |  |  |  |  |  |  |  |
| AR (1) p-values | 0.004*** | 0.005*** | 0.004*** | 0.003*** | 0.000*** | 0.004*** | 0.005*** | 0.004*** | 0.003*** | 0.000*** | 0.005*** | 0.006*** | 0.003*** | 0.004*** | 0.000*** |
| AR (2) p-values | 0.455 | 0.601 | 0.629 | 0.617 | 0.594 | 0.455 | 0.601 | 0.629 | 0.617 | 0.594 | 0.547 | 0.631 | 0.669 | 0.657 | 0.634 |
| Harsen test p-values | 0.568 | 0.584 | 0.597 | 0.609 | 0.577 | 0.568 | 0.584 | 0.597 | 0.609 | 0.577 | 0.570 | 0.566 | 0.579 | 0.591 | 0.559 |

Note: Data sources and definitions for all variables are provided in Table 1

Standard errors in parentheses

*** p<0.01, ** p<0.05, * p<0.1

**Table E1: The impact of governance and capital flows on average dietary energy supply adequacy (Difference GMM estimates)**

| Dep. Variable: Average Dietary Energy Supply Adequacy (ADESA) | (1) | (2) | (3) | (4) | (5) | (6) | (7) | (8) | (9) | (10) | (11) | (12) | (13) | (14) | (15) |
| --- | --- | --- | --- | --- | --- | --- | --- | --- | --- | --- | --- | --- | --- | --- | --- |
| Lagged dep. Variable(t-1) | 0.914*** | 0.904*** | 0.916*** | 0.913*** | 0.900*** | 0.936*** | 0.927*** | 0.938*** | 0.935*** | 0.923*** | 0.935*** | 0.940*** | 0.936*** | 0.940*** | 0.935*** |
|  | (0.017) | (0.018) | (0.018) | (0.018) | (0.018) | (0.017) | (0.017) | (0.017) | (0.017) | (0.017) | (0.017) | (0.017) | (0.017) | (0.017) | (0.017) |
| Voice and accountability score | -0.001 | -0.001 | -0.001 | -0.001 | -0.001 |  |  |  |  |  |  |  |  |  |  |
|  | (0.001) | (0.001) | (0.001) | (0.001) | (0.001) |  |  |  |  |  |  |  |  |  |  |
| Political stability score | 0.004*** | 0.004*** | 0.004*** | 0.004*** | 0.004*** |  |  |  |  |  |  |  |  |  |  |
|  | (0.001) | (0.001) | (0.001) | (0.001) | (0.001) |  |  |  |  |  |  |  |  |  |  |
| Government effectiveness score | 0.002* | 0.001* | 0.001* | 0.001* | 0.001* |  |  |  |  |  |  |  |  |  |  |
|  | (0.001) | (0.001) | (0.001) | (0.001) | (0.001) |  |  |  |  |  |  |  |  |  |  |
| Regulatory quality score | 0.000 | 0.000 | 0.000 | 0.000 | 0.000 |  |  |  |  |  |  |  |  |  |  |
|  | (0.001) | (0.001) | (0.001) | (0.001) | (0.001) |  |  |  |  |  |  |  |  |  |  |
| Rule of law score | -0.004*** | -0.004*** | -0.004*** | -0.004*** | -0.004*** |  |  |  |  |  |  |  |  |  |  |
|  | (0.001) | (0.001) | (0.001) | (0.001) | (0.001) |  |  |  |  |  |  |  |  |  |  |
| Control of corruption score | 0.038*** | 0.038*** | 0.037*** | 0.037*** | 0.038*** |  |  |  |  |  |  |  |  |  |  |
|  | (0.007) | (0.007) | (0.007) | (0.007) | (0.007) |  |  |  |  |  |  |  |  |  |  |
| Composite governance index (CGI) |  |  |  |  |  | 0.006*** | 0.006*** | 0.006*** | 0.006*** | 0.005** |  |  |  |  |  |
|  |  |  |  |  |  | (0.002) | (0.002) | (0.002) | (0.002) | (0.002) |  |  |  |  |  |
| Foreign Direct Investment (FDI) | 0.024*** |  |  |  |  | 0.030*** |  |  |  |  |  |  |  |  |  |
|  | (0.007) |  |  |  |  | (0.010) |  |  |  |  |  |  |  |  |  |
| Portfolio Equity (PE) |  | 0.020 |  |  |  |  | 0.018 |  |  |  |  |  |  |  |  |
|  |  | (0.014) |  |  |  |  | (0.0013) |  |  |  |  |  |  |  |  |
| Official Development Assistance |  |  | 0.003* |  |  |  |  | 0.002** |  |  |  |  |  |  |  |
| (ODA) |  |  | (0.002) |  |  |  |  | (0.001) |  |  |  |  |  |  |  |
| Remittances |  |  |  | 0.000 |  |  |  |  | 0.000 |  |  |  |  |  |  |
|  |  |  |  | (0.000) |  |  |  |  | (0.000) |  |  |  |  |  |  |
| Capital flows (CF) |  |  |  |  | 0.020*** |  |  |  |  | 0.028** |  |  |  |  |  |
|  |  |  |  |  | (0.006) |  |  |  |  | (0.011) |  |  |  |  |  |
| CGI x FDI |  |  |  |  |  |  |  |  |  |  | 0.060*** |  |  |  |  |
|  |  |  |  |  |  |  |  |  |  |  | (0.009) |  |  |  |  |
| CGI x PE |  |  |  |  |  |  |  |  |  |  |  | 0.000 |  |  |  |
|  |  |  |  |  |  |  |  |  |  |  |  | (0.001) |  |  |  |
| CGI x ODA |  |  |  |  |  |  |  |  |  |  |  |  | 0.030*** |  |  |
|  |  |  |  |  |  |  |  |  |  |  |  |  | (0.009) |  |  |
| CGI x Remittances |  |  |  |  |  |  |  |  |  |  |  |  |  | 0.001** |  |
|  |  |  |  |  |  |  |  |  |  |  |  |  |  | (0.000) |  |
| CGI x CF |  |  |  |  |  |  |  |  |  |  |  |  |  |  | 0.042** |
|  |  |  |  |  |  |  |  |  |  |  |  |  |  |  | (0.021) |
| Inflation | -0.000 | -0.000 | -0.000 | -0.000 | 0.000 | 0.000 | 0.000 | -0.000 | -0.000 | 0.000 | -0.000 | -0.000 | -0.000 | -0.000 | -0.000 |
|  | (0.001) | (0.001) | (0.001) | (0.001) | (0.001) | (0.001) | (0.001) | (0.001) | (0.001) | (0.001) | (0.001) | (0.001) | (0.001) | (0.001) | (0.001) |
| Share of agriculture in GDP | 0.005** | 0.005** | 0.004* | 0.004* | 0.005** | 0.006*** | 0.006*** | 0.006*** | 0.006*** | 0.007** | 0.006*** | 0.006*** | 0.007** | 0.006*** | 0.007** |
|  | (0.002) | (0.002) | (0.002) | (0.002) | (0.002) | (0.002) | (0.002) | (0.002) | (0.002) | (0.003) | (0.002) | (0.002) | (0.003) | (0.002) | (0.003) |
| Secondary school enrolment | 0.016*** | 0.015*** | 0.017*** | 0.018*** | 0.018*** | 0.016*** | 0.015*** | 0.017*** | 0.017*** | 0.015*** | 0.017*** | 0.016*** | 0.017*** | 0.016*** | 0.016*** |
|  | (0.003) | (0.003) | (0.004) | (0.004) | (0.003) | (0.003) | (0.003) | (0.004) | (0.003) | (0.003) | (0.003) | (0.003) | (0.003) | (0.003) | (0.003) |
| Population growth | -0.009* | -0.008 | -0.007 | -0.006 | -0.007 | -0.011** | -0.009* | -0.009* | -0.008 | -0.009* | -0.009* | -0.009* | -0.009* | -0.008 | -0.009* |
|  | (0.005) | (0.005) | (0.005) | (0.005) | (0.005) | (0.005) | (0.005) | (0.005) | (0.005) | (0.005) | (0.005) | (0.005) | (0.005) | (0.005) | (0.005) |
| Constant | 0.427*** | 0.479*** | 0.414*** | 0.443*** | 0.483*** | 0.333*** | 0.381*** | 0.317*** | 0.347*** | 0.388*** | 0.348*** | 0.331*** | 0.341*** | 0.328*** | 0.346*** |
|  | (0.075) | (0.076) | (0.076) | (0.075) | (0.075) | (0.073) | (0.074) | (0.074) | (0.073) | (0.073) | (0.073) | (0.072) | (0.073) | (0.072) | (0.072) |
| Observations | 375 | 375 | 375 | 375 | 375 | 375 | 375 | 375 | 375 | 375 | 375 | 375 | 375 | 375 | 375 |
| Number of countries | 25 | 25 | 25 | 25 | 25 | 25 | 25 | 25 | 25 | 25 | 25 | 25 | 25 | 25 | 25 |
| Test (p-values) |  |  |  |  |  |  |  |  |  |  |  |  |  |  |  |
| AR (1) p-values | 0.001*** | 0.002*** | 0.002*** | 0.001*** | 0.001*** | 0.001*** | 0.002*** | 0.002*** | 0.001*** | 0.001*** | 0.001*** | 0.001*** | 0.001*** | 0.001*** | 0.001*** |
| AR (2) p-values | 0.551 | 0.618 | 0.645 | 0.633 | 0.610 | 0.551 | 0.618 | 0.645 | 0.633 | 0.610 | 0.494 | 0.455 | 0.477 | 0.465 | 0.442 |
| Harsen test p-values | 0.608 | 0.576 | 0.608 | 0.620 | 0.588 | 0.608 | 0.576 | 0.608 | 0.620 | 0.588 | 0.556 | 0.533 | 0.545 | 0.557 | 0.525 |

Note: Data sources and definitions for all variables are provided in Table 1

Standard errors in parentheses

*** p<0.01, ** p<0.05, * p<0.1

**Table E2: The impact of governance and capital flows on average dietary energy supply adequacy (System GMM estimates)**

| Dep. Variable: Average Dietary Energy Supply Adequacy (ADESA) | (1) | (2) | (3) | (4) | (5) | (6) | (7) | (8) | (9) | (10) | (11) | (12) | (13) | (14) | (15) |
| --- | --- | --- | --- | --- | --- | --- | --- | --- | --- | --- | --- | --- | --- | --- | --- |
| Lagged dep. Variable(t-1) | 1.022*** | 1.023*** | 1.023*** | 1.015*** | 1.019*** | 1.033*** | 1.034*** | 1.033*** | 1.026*** | 1.029*** | 1.032*** | 1.045*** | 1.033*** | 1.036*** | 1.036*** |
|  | (0.031) | (0.014) | (0.014) | (0.014) | (0.014) | (0.013) | (0.014) | (0.014) | (0.014) | (0.014) | (0.014) | (0.013) | (0.014) | (0.013) | (0.014) |
| Voice and accountability score | -0.000 | -0.000 | -0.000 | -0.000 | -0.000 |  |  |  |  |  |  |  |  |  |  |
|  | (0.002) | (0.001) | (0.001) | (0.001) | (0.001) |  |  |  |  |  |  |  |  |  |  |
| Political stability score | 0.006*** | 0.006*** | 0.006*** | 0.005*** | 0.006*** |  |  |  |  |  |  |  |  |  |  |
|  | (0.002) | (0.001) | (0.001) | (0.001) | (0.001) |  |  |  |  |  |  |  |  |  |  |
| Government effectiveness score | 0.002 | 0.002*** | 0.002*** | 0.003*** | 0.002*** |  |  |  |  |  |  |  |  |  |  |
|  | (0.002) | (0.001) | (0.001) | (0.001) | (0.001) |  |  |  |  |  |  |  |  |  |  |
| Regulatory quality score | -0.002 | -0.002* | -0.001* | -0.001* | -0.002* |  |  |  |  |  |  |  |  |  |  |
|  | (0.001) | (0.001) | (0.001) | (0.001) | (0.001) |  |  |  |  |  |  |  |  |  |  |
| Rule of law score | -0.007*** | -0.007*** | -0.007*** | -0.008*** | -0.007*** |  |  |  |  |  |  |  |  |  |  |
|  | (0.003) | (0.001) | (0.001) | (0.001) | (0.001) |  |  |  |  |  |  |  |  |  |  |
| Control of corruption score | 0.025* | 0.025*** | 0.025*** | 0.026*** | 0.025*** |  |  |  |  |  |  |  |  |  |  |
|  | (0.014) | (0.006) | (0.006) | (0.006) | (0.006) |  |  |  |  |  |  |  |  |  |  |
| Composite governance index (CGI) |  |  |  |  |  | 0.005** | 0.005** | 0.005** | 0.004** | 0.006*** |  |  |  |  |  |
|  |  |  |  |  |  | (0.002) | (0.002) | (0.002) | (0.002) | (0.002) |  |  |  |  |  |
| Foreign Direct Investment (FDI) | 0.030*** |  |  |  |  | 0.034*** |  |  |  |  |  |  |  |  |  |
|  | (0.008) |  |  |  |  | (0.012) |  |  |  |  |  |  |  |  |  |
| Portfolio Equity (PE) |  | 0.025 |  |  |  |  | 0.026* |  |  |  |  |  |  |  |  |
|  |  | (0.018) |  |  |  |  | (0.016) |  |  |  |  |  |  |  |  |
| Official Development Assistance (ODA) |  |  | 0.004*** |  |  |  |  | 0.025*** |  |  |  |  |  |  |  |
|  |  |  | (0.001) |  |  |  |  | (0.002) |  |  |  |  |  |  |  |
| Remittances |  |  |  | 0.001** |  |  |  |  | 0.002*** |  |  |  |  |  |  |
|  |  |  |  | (0.000) |  |  |  |  | (0.000) |  |  |  |  |  |  |
| Capital flows (CF) |  |  |  |  | 0.020*** |  |  |  |  | 0.024** |  |  |  |  |  |
|  |  |  |  |  | (0.006) |  |  |  |  | (0.005) |  |  |  |  |  |
| CGI x FDI |  |  |  |  |  |  |  |  |  |  | 0.068* |  |  |  |  |
|  |  |  |  |  |  |  |  |  |  |  | (0.035) |  |  |  |  |
| CGI x PE |  |  |  |  |  |  |  |  |  |  |  | 0.000* |  |  |  |
|  |  |  |  |  |  |  |  |  |  |  |  | (0.000) |  |  |  |
| CGI x ODA |  |  |  |  |  |  |  |  |  |  |  |  | 0.050*** |  |  |
|  |  |  |  |  |  |  |  |  |  |  |  |  | (0.010) |  |  |
| CGI x Remittances |  |  |  |  |  |  |  |  |  |  |  |  |  | 0.002** |  |
|  |  |  |  |  |  |  |  |  |  |  |  |  |  | (0.000) |  |
| CGI x CF |  |  |  |  |  |  |  |  |  |  |  |  |  |  | 0.062*** |
|  |  |  |  |  |  |  |  |  |  |  |  |  |  |  | (0.023) |
| Inflation | -0.001 | -0.001* | -0.001* | -0.001 | -0.001 | -0.001 | -0.001 | -0.001 | -0.000 | -0.001 | -0.001 | -0.001 | -0.001 | -0.001 | -0.001 |
|  | (0.001) | (0.001) | (0.001) | (0.001) | (0.001) | (0.001) | (0.001) | (0.001) | (0.001) | (0.001) | (0.001) | (0.001) | (0.001) | (0.001) | (0.001) |
| Share of agriculture in GDP | 0.007** | 0.006** | 0.007** | 0.007** | 0.007** | 0.008*** | 0.008*** | 0.009*** | 0.008*** | 0.008*** | 0.008*** | 0.008*** | 0.008*** | 0.008*** | 0.008*** |
|  | (0.003) | (0.003) | (0.003) | (0.003) | (0.003) | (0.003) | (0.003) | (0.003) | (0.003) | (0.003) | (0.003) | (0.003) | (0.003) | (0.003) | (0.003) |
| Secondary school enrolment | 0.030*** | 0.030*** | 0.031*** | 0.039*** | 0.032*** | 0.029*** | 0.029*** | 0.029*** | 0.037*** | 0.030*** | 0.029*** | 0.030*** | 0.029*** | 0.030*** | 0.030*** |
|  | (0.011) | (0.003) | (0.003) | (0.003) | (0.003) | (0.003) | (0.003) | (0.003) | (0.003) | (0.003) | (0.003) | (0.003) | (0.003) | (0.003) | (0.003) |
| Population growth | -0.049* | -0.049*** | -0.049*** | -0.051*** | -0.050*** | -0.046*** | -0.046*** | -0.046*** | -0.048*** | -0.047*** | -0.046*** | -0.045*** | -0.046*** | -0.047*** | -0.046*** |
|  | (0.030) | (0.004) | (0.004) | (0.005) | (0.004) | (0.004) | (0.004) | (0.004) | (0.004) | (0.004) | (0.004) | (0.004) | (0.004) | (0.004) | (0.004) |
| Constant | 0.321*** | 0.374*** | 0.351*** | 0.322*** | 0.361*** | 0.234*** | 0.257*** | 0.272*** | 0.264*** | 0.268*** | 0.314*** | 0.361*** | 0.355*** | 0.366*** | 0.356*** |
|  | (0.150) | (0.060) | (0.060) | (0.062) | (0.059) | (0.057) | (0.058) | (0.058) | (0.059) | (0.057) | (0.058) | (0.057) | (0.057) | (0.057) | (0.057) |
| Observations | 400 | 400 | 400 | 400 | 400 | 400 | 400 | 400 | 400 | 400 | 400 | 400 | 400 | 400 | 400 |
| Number of countries | 25 | 25 | 25 | 25 | 25 | 25 | 25 | 25 | 25 | 25 | 25 | 25 | 25 | 25 | 25 |
| Test (p-values) |  |  |  |  |  |  |  |  |  |  |  |  |  |  |  |
| AR (1) p-values | 0.001*** | 0.003*** | 0.001*** | 0.001*** | 0.001*** | 0.001*** | 0.003*** | 0.001*** | 0.001*** | 0.001*** | 0.001*** | 0.001*** | 0.002*** | 0.002*** | 0.001*** |
| AR (2) p-values | 0.381 | 0.163 | 0.201 | 0.189 | 0.166 | 0.381 | 0.163 | 0.201 | 0.189 | 0.166 | 0.498 | 0.51 | 0.472 | 0.458 | 0.475 |
| Harsen test p-values | 0.624 | 0.564 | 0.577 | 0.589 | 0.557 | 0.624 | 0.564 | 0.577 | 0.589 | 0.557 | 0.579 | 0.567 | 0.554 | 0.607 | 0.547 |

Note: Data sources and definitions for all variables are provided in Table 1

Standard errors in parentheses



*** p<0.01, ** p<0.05, * p<0.1

**Table F1: The impact of governance and capital flows on undernourishment (Difference GMM estimates)**

| Dep. Variable: Undernourishment | (1) | (2) | (3) | (4) | (5) | (6) | (7) | (8) | (9) | (10) | (11) | (12) | (13) | (14) | (15) |
| --- | --- | --- | --- | --- | --- | --- | --- | --- | --- | --- | --- | --- | --- | --- | --- |
| Lagged dep. Variable(t-1) | 0.878*** | 0.880*** | 0.880*** | 0.880*** | 0.880*** | 0.898*** | 0.900*** | 0.900*** | 0.900*** | 0.900*** | 0.894*** | 0.892*** | 0.897*** | 0.903*** | 0.896*** |
|  | (0.033) | (0.034) | (0.034) | (0.034) | (0.034) | (0.032) | (0.032) | (0.032) | (0.032) | (0.032) | (0.032) | (0.032) | (0.032) | (0.033) | (0.032) |
| Voice and accountability score | 0.005 | 0.005 | 0.006 | 0.005 | 0.005 |  |  |  |  |  |  |  |  |  |  |
|  | (0.007) | (0.007) | (0.007) | (0.007) | (0.007) |  |  |  |  |  |  |  |  |  |  |
| Political stability score | -0.013* | -0.014** | -0.014** | -0.014* | -0.014** |  |  |  |  |  |  |  |  |  |  |
|  | (0.007) | (0.007) | (0.007) | (0.007) | (0.007) |  |  |  |  |  |  |  |  |  |  |
| Government effectiveness score | -0.008 | -0.008 | -0.008 | -0.008 | -0.008 |  |  |  |  |  |  |  |  |  |  |
|  | (0.005) | (0.005) | (0.005) | (0.005) | (0.005) |  |  |  |  |  |  |  |  |  |  |
| Regulatory quality score  | 0.003 | 0.004 | 0.004 | 0.004 | 0.004 |  |  |  |  |  |  |  |  |  |  |
|  | (0.005) | (0.005) | (0.005) | (0.005) | (0.005) |  |  |  |  |  |  |  |  |  |  |
| Rule of law score | -0.003 | -0.003 | -0.003 | -0.002 | -0.003 |  |  |  |  |  |  |  |  |  |  |
|  | (0.007) | (0.007) | (0.007) | (0.007) | (0.007) |  |  |  |  |  |  |  |  |  |  |
| Control of corruption score | -0.075 | -0.067 | -0.068 | -0.070 | -0.070 |  |  |  |  |  |  |  |  |  |  |
|  | (0.046) | (0.046) | (0.046) | (0.046) | (0.046) |  |  |  |  |  |  |  |  |  |  |
| Composite governance index |  |  |  |  |  | -0.047*** | -0.050*** | -0.048*** | -0.048*** | -0.049*** |  |  |  |  |  |
| (CGI) |  |  |  |  |  | (0.015) | (0.015) | (0.015) | (0.015) | (0.015) |  |  |  |  |  |
| Foreign Direct Investment (FDI) | -0.001 |  |  |  |  | -0.001 |  |  |  |  |  |  |  |  |  |
|  | (0.001) |  |  |  |  | (0.001) |  |  |  |  |  |  |  |  |  |
| Portfolio Equity (PE) |  | 0.000 |  |  |  |  | 0.001 |  |  |  |  |  |  |  |  |
|  |  | (0.001) |  |  |  |  | (0.001) |  |  |  |  |  |  |  |  |
| Official Development Assistance |  |  | 0.001 |  |  |  |  | 0.001 |  |  |  |  |  |  |  |
| (ODA) |  |  | (0.003) |  |  |  |  | (0.003) |  |  |  |  |  |  |  |
| Remittances  |  |  |  | -0.002** |  |  |  |  | -0.002** |  |  |  |  |  |  |
|  |  |  |  | (0.000) |  |  |  |  | (0.000) |  |  |  |  |  |  |
| Capital flows (CF) |  |  |  |  | -0.001* |  |  |  |  | -0.002** |  |  |  |  |  |
|  |  |  |  |  | (0.000) |  |  |  |  | (0.0001) |  |  |  |  |  |
| CGI x FDI |  |  |  |  |  |  |  |  |  |  | -0.001* |  |  |  |  |
|  |  |  |  |  |  |  |  |  |  |  | (0.001) |  |  |  |  |
| CGI x PE |  |  |  |  |  |  |  |  |  |  |  | -0.000 |  |  |  |
|  |  |  |  |  |  |  |  |  |  |  |  | (0.001) |  |  |  |
| CGI x ODA |  |  |  |  |  |  |  |  |  |  |  |  | -0.002*** |  |  |
|  |  |  |  |  |  |  |  |  |  |  |  |  | (0.001) |  |  |
| CGI x Remittances |  |  |  |  |  |  |  |  |  |  |  |  |  | -0.002*** |  |
|  |  |  |  |  |  |  |  |  |  |  |  |  |  | (0.001) |  |
| CGI x CF |  |  |  |  |  |  |  |  |  |  |  |  |  |  | -0.021** |
|  |  |  |  |  |  |  |  |  |  |  |  |  |  |  | (0.010) |
| Inflation | -0.000 | 0.000 | 0.000 | -0.000 | 0.000 | -0.001 | -0.000 | -0.001 | -0.001 | -0.001 | -0.000 | -0.000 | -0.000 | -0.001 | -0.001 |
|  | (0.005) | (0.005) | (0.005) | (0.005) | (0.005) | (0.005) | (0.005) | (0.005) | (0.005) | (0.005) | (0.005) | (0.005) | (0.005) | (0.005) | (0.005) |
| Share of agriculture in GDP | 0.051*** | 0.051*** | 0.050*** | 0.053*** | 0.051*** | 0.051*** | 0.052*** | 0.052*** | 0.054*** | 0.052*** | 0.054*** | 0.054*** | 0.054*** | 0.055*** | 0.054*** |
|  | (0.018) | (0.018) | (0.018) | (0.018) | (0.018) | (0.018) | (0.018) | (0.018) | (0.018) | (0.018) | (0.018) | (0.018) | (0.018) | (0.018) | (0.018) |
| Secondary school enrolment | -0.031** | -0.028** | -0.029** | -0.039** | -0.031** | -0.029** | -0.026* | -0.029* | -0.039*** | -0.029** | -0.031** | -0.035*** | -0.031** | -0.036*** | -0.032*** |
|  | (0.014) | (0.014) | (0.014) | (0.016) | (0.015) | (0.013) | (0.014) | (0.014) | (0.015) | (0.014) | (0.013) | (0.013) | (0.013) | (0.013) | (0.013) |
| Population growth | 0.074* | 0.077** | 0.079** | 0.083** | 0.078** | 0.053 | 0.053 | 0.055 | 0.060 | 0.054 | 0.071* | 0.088** | 0.061 | 0.069* | 0.067* |
|  | (0.038) | (0.038) | (0.038) | (0.039) | (0.038) | (0.038) | (0.038) | (0.038) | (0.038) | (0.038) | (0.037) | (0.037) | (0.038) | (0.037) | (0.038) |
| Constant | 0.508*** | 0.480*** | 0.464** | 0.487*** | 0.484*** | 0.440** | 0.412** | 0.410** | 0.423** | 0.413** | 0.429** | 0.423** | 0.414** | 0.392** | 0.419** |
|  | (0.182) | (0.181) | (0.187) | (0.182) | (0.182) | (0.179) | (0.178) | (0.184) | (0.179) | (0.179) | (0.178) | (0.178) | (0.178) | (0.178) | (0.178) |
| Observations | 375 | 375 | 375 | 375 | 375 | 375 | 375 | 375 | 375 | 375 | 375 | 375 | 375 | 375 | 375 |
| Number of countries | 25 | 25 | 25 | 25 | 25 | 25 | 25 | 25 | 25 | 25 | 25 | 25 | 25 | 25 | 25 |
| Test (p-values) |  |  |  |  |  |  |  |  |  |  |  |  |  |  |  |
| AR (1) p-values | 0.000*** | 0.000*** | 0.001*** | 0.000*** | 0.000*** | 0.000*** | 0.000*** | 0.001*** | 0.000*** | 0.000*** | 0.000*** | 0.000*** | 0.001*** | 0.000*** | 0.000*** |
| AR (2) p-values | 0.548 | 0.232 | 0.259 | 0.247 | 0.224 | 0.548 | 0.232 | 0.259 | 0.247 | 0.224 | 0.460 | 0.377 | 0.404 | 0.392 | 0.369 |
| Harsen test p-values | 0.519 | 0.600 | 0.631 | 0.643 | 0.611 | 0.519 | 0.600 | 0.631 | 0.643 | 0.611 | 0.520 | 0.513 | 0.535 | 0.547 | 0.515 |

Note: Data sources and definitions for all variables are provided in Table 1

Standard errors in parentheses

*** p<0.01, ** p<0.05, * p<0.1

**Table F2: The impact of governance and capital flows on undernourishment (System GMM estimates)**

| Dep. Variable: Undernourishment | (1) | (2) | (3) | (4) | (5) | (6) | (7) | (8) | (9) | (10) | (11) | (12) | (13) | (14) | (15) |
| --- | --- | --- | --- | --- | --- | --- | --- | --- | --- | --- | --- | --- | --- | --- | --- |
| Lagged dep. Variable(t-1) | 0.906*** | 0.910*** | 0.908*** | 0.903*** | 0.910*** | 0.911*** | 0.917*** | 0.913*** | 0.907*** | 0.917*** | 0.904*** | 0.898*** | 0.906*** | 0.910*** | 0.903*** |
|  | (0.022) | (0.022) | (0.022) | (0.022) | (0.022) | (0.021) | (0.021) | (0.022) | (0.021) | (0.022) | (0.021) | (0.021) | (0.021) | (0.021) | (0.021) |
| Voice and accountability score | 0.000 | 0.000 | 0.000 | 0.000 | 0.000 |  |  |  |  |  |  |  |  |  |  |
|  | (0.007) | (0.007) | (0.007) | (0.007) | (0.007) |  |  |  |  |  |  |  |  |  |  |
| Political stability score | -0.014** | -0.015** | -0.015** | -0.012* | -0.015** |  |  |  |  |  |  |  |  |  |  |
|  | (0.007) | (0.007) | (0.007) | (0.007) | (0.007) |  |  |  |  |  |  |  |  |  |  |
| Government effectiveness score | -0.007 | -0.007 | -0.007 | -0.007 | -0.007 |  |  |  |  |  |  |  |  |  |  |
|  | (0.005) | (0.005) | (0.005) | (0.005) | (0.005) |  |  |  |  |  |  |  |  |  |  |
| Regulatory quality score | 0.004 | 0.004 | 0.005 | 0.004 | 0.005 |  |  |  |  |  |  |  |  |  |  |
|  | (0.005) | (0.005) | (0.005) | (0.005) | (0.005) |  |  |  |  |  |  |  |  |  |  |
| Rule of law score | -0.005 | -0.006 | -0.005 | -0.005 | -0.006 |  |  |  |  |  |  |  |  |  |  |
|  | (0.007) | (0.007) | (0.007) | (0.007) | (0.007) |  |  |  |  |  |  |  |  |  |  |
| Control of corruption score | -0.150*** | -0.138*** | -0.147*** | -0.157*** | -0.140*** |  |  |  |  |  |  |  |  |  |  |
|  | (0.039) | (0.039) | (0.039) | (0.039) | (0.040) |  |  |  |  |  |  |  |  |  |  |
| Composite governance index |  |  |  |  |  | -0.061*** | -0.062*** | -0.060*** | -0.065*** | -0.061*** |  |  |  |  |  |
| (CGI) |  |  |  |  |  | (0.013) | (0.013) | (0.013) | (0.013) | (0.013) |  |  |  |  |  |
| Foreign Direct Investment (FDI) | -0.001* |  |  |  |  | -0.001* |  |  |  |  |  |  |  |  |  |
|  | (0.001) |  |  |  |  | (0.001) |  |  |  |  |  |  |  |  |  |
| Portfolio Equity (PE) |  | 0.001 |  |  |  |  | -0.001 |  |  |  |  |  |  |  |  |
|  |  | (0.001) |  |  |  |  | (0.001) |  |  |  |  |  |  |  |  |
| Official Development Assistance |  |  | -0.002* |  |  |  |  | -0.002** |  |  |  |  |  |  |  |
| (ODA) |  |  | (0.001) |  |  |  |  | (0.001) |  |  |  |  |  |  |  |
| Remittances |  |  |  | -0.004** |  |  |  |  | -0.005*** |  |  |  |  |  |  |
|  |  |  |  | (0.002) |  |  |  |  | (0.002) |  |  |  |  |  |  |
| Capital flows (CF) |  |  |  |  | -0.002** |  |  |  |  | -0.003*** |  |  |  |  |  |
|  |  |  |  |  | (0.001) |  |  |  |  | (0.001) |  |  |  |  |  |
| CGI x FDI |  |  |  |  |  |  |  |  |  |  | -0.002*** |  |  |  |  |
|  |  |  |  |  |  |  |  |  |  |  | (0.001) |  |  |  |  |
| CGI x PE |  |  |  |  |  |  |  |  |  |  |  | -0.000 |  |  |  |
|  |  |  |  |  |  |  |  |  |  |  |  | (0.000) |  |  |  |
| CGI x ODA |  |  |  |  |  |  |  |  |  |  |  |  | -0.003*** |  |  |
|  |  |  |  |  |  |  |  |  |  |  |  |  | (0.001) |  |  |
| CGI x Remittances |  |  |  |  |  |  |  |  |  |  |  |  |  | -0.003*** |  |
|  |  |  |  |  |  |  |  |  |  |  |  |  |  | (0.001) |  |
| CGI x CF |  |  |  |  |  |  |  |  |  |  |  |  |  |  | -0.033** |
|  |  |  |  |  |  |  |  |  |  |  |  |  |  |  | (0.015) |
| Inflation | 0.001 | 0.002 | 0.001 | 0.001 | 0.002 | 0.001 | 0.002 | 0.001 | -0.000 | 0.001 | 0.001 | 0.002 | 0.001 | 0.001 | 0.001 |
|  | (0.005) | (0.005) | (0.005) | (0.005) | (0.005) | (0.005) | (0.005) | (0.005) | (0.005) | (0.005) | (0.004) | (0.004) | (0.004) | (0.005) | (0.005) |
| Share of agriculture in GDP | 0.060*** | 0.059*** | 0.060*** | 0.063*** | 0.059*** | 0.057*** | 0.057*** | 0.057*** | 0.060*** | 0.056*** | 0.056*** | 0.053*** | 0.057*** | 0.057*** | 0.057*** |
|  | (0.016) | (0.016) | (0.016) | (0.016) | (0.016) | (0.016) | (0.016) | (0.016) | (0.016) | (0.016) | (0.016) | (0.016) | (0.016) | (0.016) | (0.016) |
| Secondary school enrolment | -0.066*** | -0.063*** | -0.066*** | -0.084*** | 0.062*** | -0.041** | -0.039** | -0.041** | -0.060*** | -0.037** | -0.042*** | -0.045*** | -0.041** | -0.044*** | -0.042*** |
|  | (0.018) | (0.018) | (0.018) | (0.020) | (0.019) | (0.016) | (0.016) | (0.016) | (0.018) | (0.016) | (0.016) | (0.016) | (0.016) | (0.016) | (0.016) |
| Population growth | 0.164*** | 0.159*** | 0.166*** | 0.154*** | -0.164*** | 0.160*** | 0.154*** | 0.163*** | 0.147*** | 0.161*** | 0.160*** | 0.161*** | 0.157*** | 0.160*** | 0.161*** |
|  | (0.023) | (0.023) | (0.023) | (0.023) | (0.023) | (0.022) | (0.022) | (0.023) | (0.023) | (0.022) | (0.022) | (0.022) | (0.022) | (0.022) | (0.022) |
| Constant | 0.426*** | 0.395*** | 0.397*** | 0.432*** | 0.384*** | 0.476*** | 0.436*** | 0.438*** | 0.497*** | 0.420*** | 0.474*** | 0.448*** | 0.474*** | 0.458*** | 0.481*** |
|  | (0.135) | (0.133) | (0.147) | (0.133) | (0.137) | (0.134) | (0.131) | (0.144) | (0.131) | (0.136) | (0.130) | (0.130) | (0.130) | (0.130) | (0.130) |
| Observations | 400 | 400 | 400 | 400 | 400 | 400 | 400 | 400 | 400 | 400 | 400 | 400 | 400 | 400 | 400 |
| Number of countries | 25 | 25 | 25 | 25 | 25 | 25 | 25 | 25 | 25 | 25 | 25 | 25 | 25 | 25 | 25 |
| Test (p-values) |  |  |  |  |  |  |  |  |  |  |  |  |  |  |  |
| AR (1) p-values | 0.000*** | 0.000*** | 0.001*** | 0.000*** | 0.000*** | 0.000*** | 0.000*** | 0.001*** | 0.000*** | 0.000*** | 0.001*** | 0.001*** | 0.001*** | 0.000*** | 0.000*** |
| AR (2) p-values | 0.578 | 0.591 | 0.629 | 0.617 | 0.594 | 0.578 | 0.591 | 0.629 | 0.617 | 0.594 | 0.574 | 0.631 | 0.669 | 0.657 | 0.634 |
| Harsen test p-values | 0.600 | 0.584 | 0.597 | 0.609 | 0.577 | 0.600 | 0.584 | 0.597 | 0.609 | 0.577 | 0.578 | 0.566 | 0.579 | 0.591 | 0.559 |

Note: Data sources and definitions for all variables are provided in Table 1

Standard errors in parentheses

*** p<0.01, ** p<0.05, * p<0.1
